# Ingenious Architecture and Coloration Generation in Enamel of Rodent Teeth

**DOI:** 10.1021/acsnano.4c00578

**Published:** 2024-04-17

**Authors:** Vesna Srot, Sophia Houari, Gregor Kapun, Birgit Bussmann, Felicitas Predel, Boštjan Pokorny, Elena Bužan, Ute Salzberger, Bernhard Fenk, Marion Kelsch, Peter A. van Aken

**Affiliations:** † Max Planck Institute for Solid State Research, Stuttgart 70569, Germany; ‡ Unité de Formation et de Recherche d’Odontologie, Université Paris Cité, Paris 75006, France; § UR2496, Biomedical Research in Odontology, Université Paris Cité, Montrouge 92120, France; ∥ National Institute of Chemistry, Ljubljana 1000, Slovenia; ⊥ Centre of Excellence on Nanoscience and Nanotechnology−Nanocenter, Ljubljana 1000, Slovenia; # Faculty of Environmental Protection, Velenje 3320, Slovenia; ∇ Slovenian Forestry Institute, Ljubljana 1000, Slovenia; ○ Faculty of Mathematics, Natural Sciences and Information Technologies, University of Primorska, Koper 6000, Slovenia

**Keywords:** teeth microstructure, ameloblasts, ferritin, Fe-rich enamel, 3D FIB-SEM tomography, analytical (S)TEM, color

## Abstract

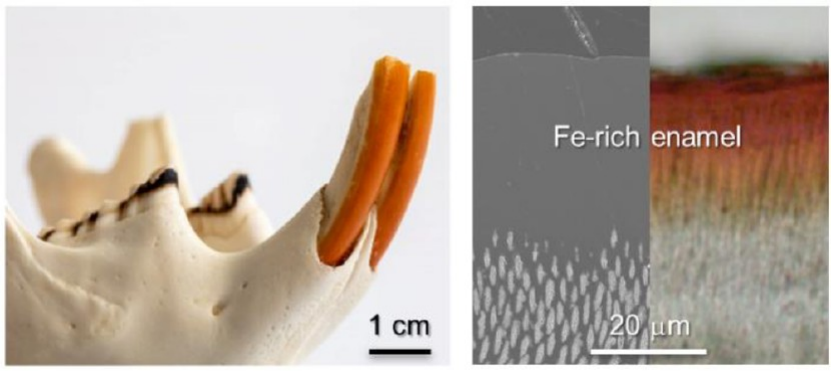

Teeth exemplify architectures comprising an interplay of inorganic and organic constituents, resulting in sophisticated natural composites. Rodents (Rodentia) showcase extraordinary adaptations, with their continuously growing incisors surpassing human teeth in functional and structural optimizations. In this study, employing state-of-the-art direct atomic-scale imaging and nanoscale spectroscopies, we present compelling evidence that the release of material from ameloblasts and the subsequent formation of iron-rich enamel and surface layers in the constantly growing incisors of rodents are complex orchestrated processes, intricately regulated and independent of environmental factors. The synergistic fusion of three-dimensional tomography and imaging techniques of etched rodent́s enamel unveils a direct correlation between the presence of pockets infused with ferrihydrite-like material and the acid resistant properties exhibited by the iron-rich enamel, fortifying it as an efficient protective shield. Moreover, observations using optical microscopy shed light on the role of iron-rich enamel as a microstructural element that acts as a path for color transmission, although the native color remains indistinguishable from that of regular enamel, challenging the prevailing paradigms. The redefinition of “pigmented enamel” to encompass ferrihydrite-like infusion in rodent incisors reshapes our perception of incisor microstructure and color generation. The functional significance of acid-resistant iron-rich enamel and the understanding of the underlying coloration mechanism in rodent incisors have far-reaching implications for human health, development of potentially groundbreaking dental materials, and restorative dentistry. These findings enable the creation of an entirely different class of dental biomaterials with enhanced properties, inspired by the ingenious designs found in nature.

Teeth are extraordinary composites that possess a diverse microstructure and exhibit exceptional physical properties.^1,2^ Among these, dental enamel stands as the hardest and most mineralized component within the hierarchical framework of teeth.^3^ Its structure is exquisitely optimized to confer long-term resistance against the turbulent fluctuations arising from various physical and chemical processes occurring within the oral cavity.^4,5^ Intriguingly, in the case of rodents (Rodentia), their constantly growing incisors display functional enhancements accompanied by structural and chemical optimizations that surpass those found in human teeth.^6−8^ Notably, the outer layer of their enamel is enriched with iron,^7,8^ serving as an insulating barrier that protects the incisors from environmental factors.^7,9^ This iron enriched enamel is known as pigmented enamel,^10−14^ possessing long-known resistance against acid attack.^12^ Numerous mammalian species exhibit the ability to continuously grow their teeth, allowing for potential structural modifications throughout their lifespan.^15^ Among these species, rodents present intriguing variations in the development and structure of their incisors and molars.^16^ The incisor enamel, in particular, undergoes a dynamic process driven by the continuous proliferation, differentiation, and transition of tooth epithelial stem cells into ameloblasts. This feature enables perpetual tooth growth and the generation of enamel, rendering rodent incisors an ideal model system for studying tooth biology.^3^

The formation and mineralization of dental enamel represent energetically demanding processes, especially in the constantly growing incisors of rodents. The sequential stages of enamel development are orchestrated by ameloblasts, which fulfill distinct functional roles.^17^ Two crucial developmental stages, namely, the secretory and maturation, are intricately linked to the evolutionary trajectory of enamel. In maturation stage ameloblasts, active ion transport, as well as the removal of matrix proteins and water necessitate a substantial production of adenosine triphosphate (ATP) through oxidative phosphorylation in numerous mitochondria. This process is aided by the energy derived from iron present in these cells.^18,19^ Structural transformations during enamel maturation result in a progressive transition and enhancement of the physicochemical properties. Soft, newly formed enamel gradually matures into a resilient, highly mineralized tissue.^3,20,21^ In rodents, the enrichment of the outer part of enamel layer with iron during the pigment release stage, called pigmentation, endows their incisors with superior functionality as self-sharpening natural tools.^1^

Iron, an indispensable element, orchestrates a multitude of essential biological functions.^22^ Its ability to exist in two thermodynamically stable oxidation states, ferric Fe^3+^ and ferrous Fe^2+^, ensures its broad accessibility within neutral aqueous environments.^22^ In mammalian organisms, the primary reservoir of iron resides in red blood cells within hemoglobin protein.^23,24^ Ferritins, universal proteins for iron storage and mineralization, comprise 24 subunits arranged in a symmetrical cubic configuration. These large spherical protein cages (∼12 nm diameter) elegantly encapsulate and govern the reversible formation of hydrated iron oxide biominerals within a central cavity (5–8 nm diameter).^25,26^ The ingress of Fe^2+^ ions occurs through the Fe^2+^ ion channels located at the 3-fold symmetry axes of the ferritin cages. The initiation of mineral formation entails the reaction of Fe^2+^ with O_2_ at specialized enzymatic sites within ferritin,^27,28^ culminating in the development of an encapsulated hydrous ferric oxide core, known as ferrihydrite (5Fe_2_O_3_·9H_2_O). The fundamental role of ferritin lies in its ability to store and accumulate biological iron in nontoxic form, a function of paramount importance for basic cellular activities.^29^ In native ferritin biomineral cores, the degree of structural order aligns with varying levels of phosphate.^30^ Mammalian ferritins with low phosphate levels exhibit high core crystallinity (Fe/P = 9–21), while noncrystalline bacterial ferritins harbor higher phosphate concentrations (Fe/P = 1.4–1.7).^29,31,32^ The exact allocation of phosphate within ferritin cores remains elusive, encompassing both surface adsorption and core incorporation. The involvement of phosphate emerges as a vital factor in iron oxide biomineralization, showcasing its versatile capacity to catalyze the biochemical reactions, potentially influencing the rate of Fe^2+^ oxidation, as well as guide the reductive dissolution of solid Fe^3+^ oxide phases.^32^ Upon reaching the enameĺs mature stage, the release and transfer of iron pigment into the enamel may occur in ionic form or attached to carrier proteins.^33,34^ Only a finite outer portion of the radial enamel becomes occupied by this iron pigment, leading to the formation of a dense, acid-resistant enamel enriched with iron, endowed with exceptional physical, chemical, and mechanical properties.^7,8,35^

## Results and Discussion

Rodents possess a dental attribute in the form of constantly growing incisors, distinguished by their specific orange-brown color (Supporting Information (SI), Figure S1). These elongated, rootless structures exemplify an ingenious constructional design, where hard enamel selectively covers the labial side of the softer bulk dentin, which results in a self-sharpening apparatus^1^ (SI, Figure S1). The fully formed enamel comprises approximately 96 wt % elongated hydroxyapatite (HA) crystals organized into rod and inter-rod enamel, while the remaining constituents consist of organic material and water.^3^ Within mature rodent enamel, the residual organic matrix, primarily composed of proteins, constitutes around 4%.^3^ Enamel structures exhibit varying degrees of complexity depending on the region (SI, Figure S2); the outer radial enamel (R-EN) is characterized by parallel arrangements of rods, whereas the inner enamel (I-EN) features rods running parallel within a single layer, with adjacent rows inclined in opposite directions.^16,36^ In this study, we embark on a comprehensive investigation, delving into the complete structural and chemical developmental trajectory of rodent incisors, spanning from the macro- to the nanoscale (SI, Figure S3). Our analysis encompasses incisors from a range of rodent species inhabiting diverse habitats, including beavers, coypus, squirrels, marmots, rats, voles, and mice. To overcome the electron beam sensitivity of the samples, we employed advanced low-voltage and low-dose (scanning) transmission electron microscopy (S)TEM imaging in conjunction with analytical characterization techniques.

In light of our current understanding, we propose a reevaluation of the conventional terminology. The previously designated “pigmented enamel” in rodent incisors, which originates from the aforementioned iron-laden enamel layer,^13,14^ contradicts our evidence. Thus, we propose the adoption of “Fe-rich enamel” (Fe-EN) as an alternative nomenclature. This reclassification aligns with our groundbreaking findings, unveiling the pivotal role of iron in the enamel layer.

### Ferritin Nanoparticles

**Figure 1:**
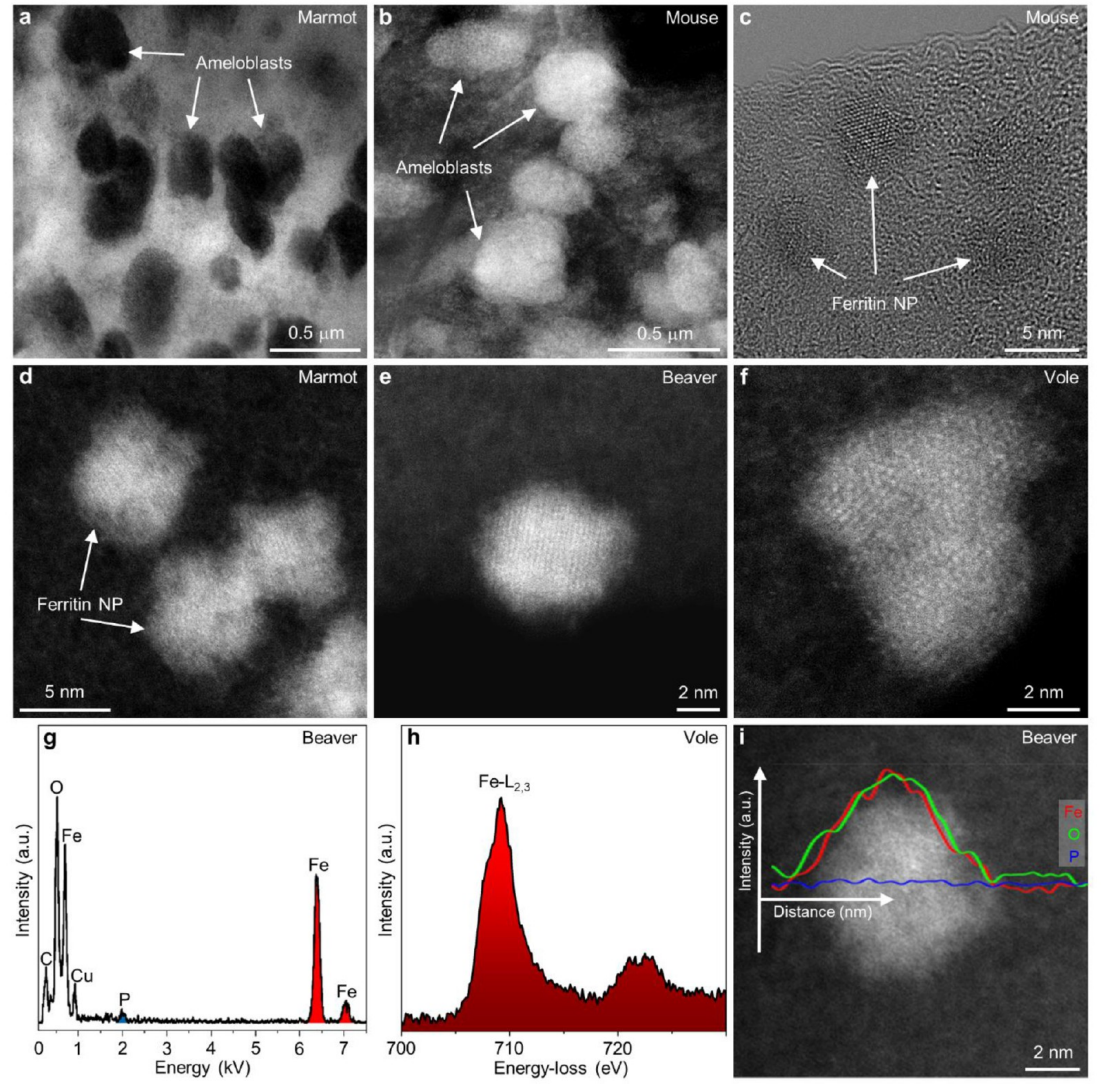
Atomic-scale structure and composition of ferritin nanoparticles in the pigmentation stage ameloblasts. (a,b) Lower magnification BF-STEM (a) and HAADF-STEM (b) images obtained from thin electron transparent sections cut through part of ameloblasts filled with ferritin nanoparticles. (c) HR-TEM image of individual crystalline ferritin nanoparticles enclosed in the organic matrix of ameloblasts. (d–f) Atomically resolved HAADF-STEM images of ferritin nanoparticles with sizes of 6–8 nm shown for different rodent species investigated. (g) EDX spectrum recorded from a single ferritin nanoparticle showing the major constituting elements Fe and O and low amounts of P. (h) Background-subtracted Fe-L_2,3_ ELNES obtained from a single ferritin nanoparticle showing iron predominantly in the 3+ oxidation state. (i) Fe, O, and P signal extracted from an EELS line-scan recorded across the single ferritin nanoparticle showing an increase in Fe and O across the particle but no obvious change in P signal.

Investigating the highly sensitive material within the pristine state of ameloblasts during the pigmentation stage demanded a changed sample preparation approach, enabling atomic resolution imaging and compositional analysis of ferritin clusters and single ferritin nanoparticles (Figure 1a–f). To mitigate potential chemical contamination and influence on composition or oxidation state, enamel organ samples were left unfixed and unstained. Lower magnification bright-field (BF)-STEM (Figure 1a) and high-angle annular dark-field (HAADF)-STEM (Figure 1b) images of electron transparent slices through ameloblasts unveil the presence of densely packed ferritin nanoparticles enclosed within the organic matrix. Consistently observed across all investigated species, these nanoparticles displayed a uniform size distribution (Figure 1c–f) with an average diameter of 6–8 nm. Atomically resolved images of single ferritin nanoparticles revealed fused core segments (Figure 1d–f), exhibiting varying orientations to each other, akin to previous observations in human hepatic ferritin.^37^ Detailed examination of ferritin core segments, oriented proximate to a crystallographic zone axis, demonstrated d-lattice spacing consistent with the crystalline structure of ferrihydrite (Figure 1c).^38^ The intense contrast of these nanoparticles in HAADF-STEM images (Figure 1b,d–f) indicated a relatively higher atomic number due to the presence of iron atoms compared to the surrounding matrix.

A robust combination of nanoscale STEM imaging and energy-dispersive X-ray (EDX) spectroscopy unveiled the relatively consistent composition of the nanosized ferritin particles, predominantly comprising Fe and O (Figure 1g). Precise compositional analysis, utilizing experimentally determined *k*-factors, revealed a low P content, resulting in an average Fe vs P ratio of 6.1–10.75 (calculated from atomic %), as measured from the single ferritin particles across all investigated species (SI, Figure S4). These values closely mirrored those reported for mammalian ferritin isolated from the liver^37,39^ and spleen.^30,31,39^ To date, no records exist of Fe/P ratios measured from single ferritin nanoparticles derived from rodent enamel organs, except for those obtained from whole ameloblasts in coypu.^8^ Because ferritin nanoparticles were not extracted from their native environment, the low P signal measured from the surrounding cytoplasm is superimposed on the total EDX signal. Therefore, the actual Fe/P ratio reported here is likely even higher. Analyzing the fine structural details of Fe-L_2,3_ energy-loss near-edge structures (ELNES) demonstrated that Fe within the ferritin nanoparticles predominantly appeared in the 3+ oxidation state (Figure 1h). Signal intensity profiles obtained across the ferritin particle, after background subtraction, from P-L_2,3_, O–K, and Fe-L_2,3_ ionization edges exhibited an increase in Fe and O originating from ferrihydrite, with no discernible fluctuations in P levels (Figure 1i).

These findings enhance our understanding and establish correlations between the microstructure and composition of ferritin mineral cores in enamel organs across different rodent species. Despite their diverse living environments, no apparent distinctions were identified. The low levels of P, resulting in crystalline ferrihydrite cores with ferric Fe, serve as a characteristic hallmark of mammalian ferritin.

### Fe-Rich Enamel

The energy demands of ameloblasts are met by iron stored in ferritin nanoparticles. During the late maturation and pigmentation stages of amelogenesis in rodent incisors, the biomineral ferrihydrite, formed inside ferritin protein cages, is dissolved and secreted out of the ameloblasts.^40^ Ferroportin proteins facilitate the export of ionic Fe^2+^ across ameloblasts apical membrane,^24^ where it undergoes transition to Fe^3+^ due to the change of pH from acidic to neutral during the process of ameloblasts modulation throughout the maturation stage.^3^

Upon enamel maturation, oxidized Fe material infiltrates the outer layer of prehardened radial enamel, occupying the vacant spaces between the HA crystals (SI, Figure S5). We observed a species-specific pattern, wherein the outer radial enamel is filled with iron-rich material to a certain depth, forming an acid resistant Fe-EN (SI, Figure S6 and Table S1). The reasons underlying the distinct structural arrangement remain unknown but may be attributed to diverse living patterns, feeding habits, gnawing behavior, genetic background, and mechanical loads. On average, the thicknesses of Fe-EN reaches approximately 15 μm (ranging from 8 μm in mice to 30 μm in coypus) (SI, Figure S6 and Table S1). Surprisingly, the thickness ratios of the constituting components in incisors remain unaltered when comparing upper and lower incisors (observed for coypu and squirrel) and even among incisors from animals of different ages (observed for coypu).

Characterizing the minuscule sizes and irregular shapes of Fe-rich pockets within Fe-EN presents exceptional challenges. To optimize the geometry for measuring individual phases without superimposition, electron transparent lamellae were fabricated in a top-view orientation, sliced parallel to the labial surface of the incisor tooth (SI, Figure S7). This approach enabled the observation of HA crystals sectioned roughly perpendicular to their long axis.

**Figure 2:**
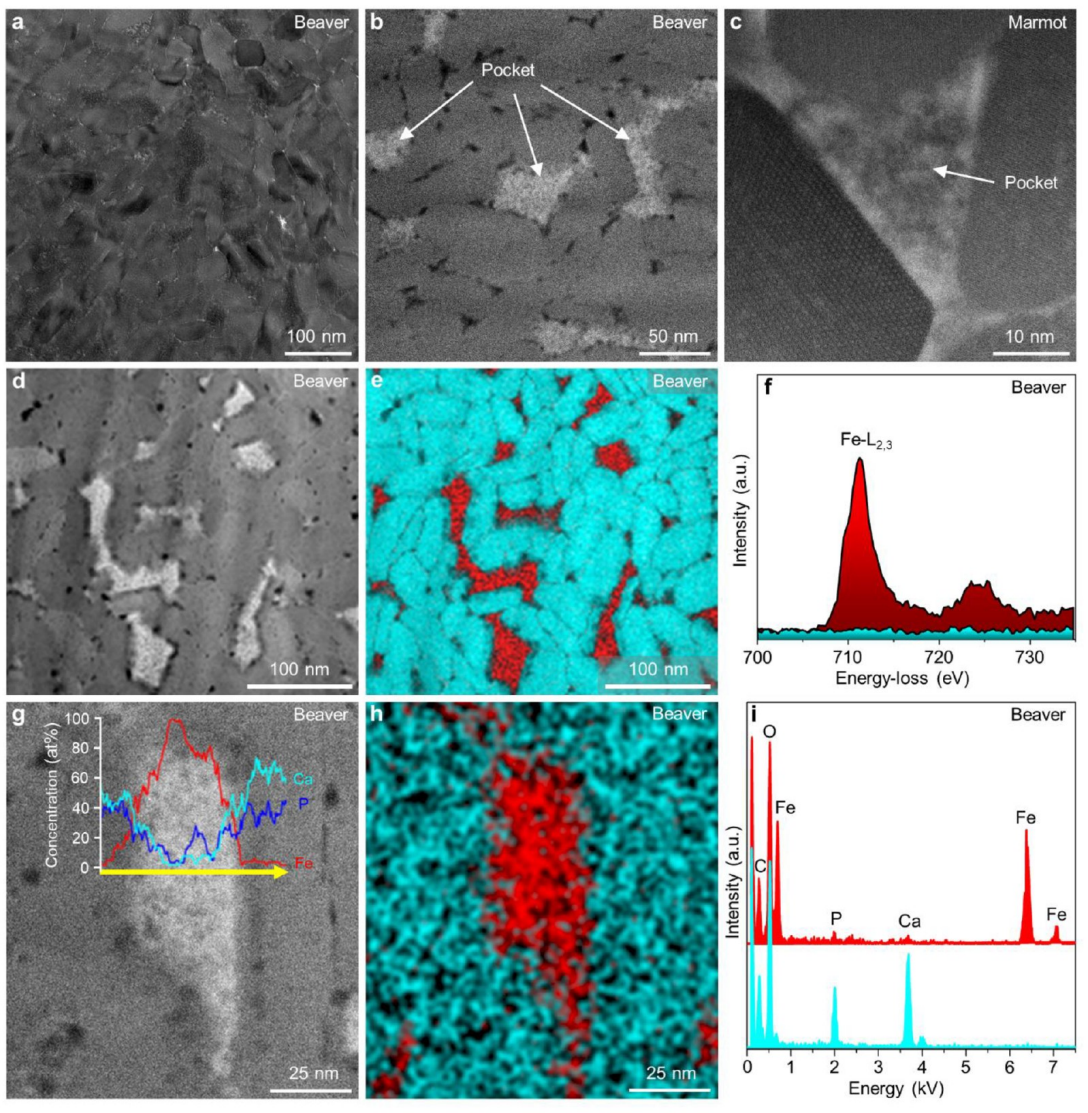
Architecture and nanoscale composition of amorphous ferrihydrite-like pockets in Fe-EN. (a) HR-TEM image of Fe-EN showing a dense structure. (b) HAADF-STEM image of ferrihydrite-like pockets surrounding HA crystals within Fe-EN. The pockets appear brighter in the image and fill the spaces between the HA crystals. (c) Atomically resolved BF-STEM image of a single ferrihydrite-like pocket surrounding HA crystals. HA are viewed perpendicular to their *c*-axis. (d,e) Lower magnification ADF-STEM image of Fe-EN with HA crystals surrounded by brighter ferrihydrite-like pockets (d) with corresponding overlapping Fe (red) and Ca (cyan) EELS elemental maps (e) obtained from background subtracted signals of Ca-M_2,3_ and Fe-M_2,3_ edges. (f) Background-subtracted Fe-L_2,3_ ELNES acquired from ferrihydrite-like material within the pockets (red). The spectrum acquired from the HA crystal in the same energy range shows no signal enhancement (cyan). (g–i) HAADF-STEM image of a ferrihydrite-like pocket in Fe-EN (g) with superimposed Fe, Ca, and P EDX line-scan signals measured across the pocket. Corresponding superimposed Ca (cyan) and Fe (red) EDX elemental maps (h) and (i) EDX spectra measured from the ferrihydrite-like pocket (red) and in the nearby HA crystal (cyan). The small Ca and P contributions in the spectrum from the ferrihydrite-like pocket (red) are attributed to the material redeposition during the TEM sample preparation and/or scattering from the nearby lying HA crystals. TEM specimens were prepared parallel to the surface of the incisor (a–i).

High-resolution transmission electron microscopy (HR-TEM) images of Fe-EN revealed a close-packed structure (Figure 2a). The HAADF-STEM image (Figure 2b) exhibited brighter contrast in the pocket material compared with adjacent HA crystals, indicating the presence of a chemically distinct phase, as expected. Cross-sectional analysis revealed an average characteristic size of approximately 50 nm across all studied species. The atomically resolved BF-STEM image of an HA crystal (Figure 2c, bottom left) in Fe-EN exhibited an elongated hexagonal cross-section with well-defined edges, corresponding to the crystal orientation normal to the long crystal axis of HA (close to the (0001) *c*-plane). The atomically sharp edges of HA crystals were in close contact with the pocket material, forming a dense Fe-EN. Fourier transformation (FT) patterns indicated that the infiltrated pocket material possessed an amorphous structure. Nanoscale chemical analysis of Fe-EN, using a combination of annular dark-field (ADF)-STEM imaging and EELS elemental mapping, demonstrated the spatial distribution of Fe-rich pockets (Figure 2d,e). The presence of a Ca signal was attributed to the HA crystals, while the Fe signal originated from the pockets. The fine structural details of the Fe-L_2,3_ ELNES unequivocally indicated the presence of Fe in the 3+ oxidation state for all of the investigated species, consistent with our expectations.

The spatial distribution and elemental composition of Fe-rich pockets within rodent incisors were investigated by using energy-dispersive X-ray spectroscopy (EDX) coupled with line profiles, elemental maps, and spectral analysis. The EDX line profiles of Fe (red), Ca (cyan), and P (blue) signals extracted from the EDX area measurement (Figure 2g) and the superimposed Fe (red) and Ca (cyan) EDX elemental maps (Figure 2h) revealed a sharp increase in Fe signal across the pocket, accompanied by a near absence of Ca and P signals in the middle of the pocket. Conversely, the HA crystals displayed no detectable Fe signal (Figure 2g,h). Furthermore, the irregular shape of the pockets was found to influence compositional fluctuations, as is evident from the EDX line-scan profiles and superimposed Fe and Ca area maps (Figure 2g,h). Although no contrast modulations were observed in the HAADF-STEM image (Figure 2g), the inclined contact between the pocket and HA crystals resulted in substantial intermixing of EDX signals, particularly on both sides of the pocket (Figure 2g). The EDX spectrum of the pure pocket material (Figure 2i, red) exhibited prominent Fe and O signals, while the spectrum of the pure HA crystal (Figure 2i, cyan) showed Ca and P signals. Our investigations, involving a comprehensive analysis of pockets from seven rodent species (comprising a total of 74 pockets), revealed a predominantly Fe oxide/oxyhydroxide (ferrihydrite-like) phase.

These results provide robust evidence of a unified developmental pattern of Fe-EN in the incisors of diverse rodent species. The amorphous ferrihydrite-like phase, forming a secondary component within the Fe-EN, occupies the originally empty spaces between the HA crystals in the outer radial enamel. The occurrence of amorphous phases during iron-oxide biomineralization, attributed to the rapid oxidation processes and inhibition of crystallization in the presence of phosphates,^32^ supports our findings. This concept, coupled with the demonstrated data, aligns with earlier results from coypu^8,35^ but contradicts the findings obtained by investigations on separate rodent species by atom probe tomography (APT) in mouse and rat.^7^ Although their qualitative comparison of Fe–K edge X-ray absorption near edge (XANES) and extended X-ray absorption fine structure (EXAFS) spectra and quantitative analysis of EXAFS data along with Mössbauer and Raman spectroscopy data in beaver incisors shows the presence of ferrihydrite, the authors proposed an intergranular phase consisting of a mixture of ferrihydrite (58%) and amorphous Fe–Ca phosphate (42%).^7^ Closer inspection of their APT concentration data shows a rather high level of Ca, two times higher than the amount of Fe, which contradicts their conclusions. Comparing the data, the intergranular phase^7^ and the pockets described here appear to be the same structural unit. We suggest that their APT concentration values result from a mixed signal from the intergranular phase and nearby positioned hydroxyapatite crystals.

### 3D Distribution of Ferrihydrite-Like Pockets

In order to gain insight into the intricate three-dimensional (3D) morphology and spatial arrangement of pockets within the Fe-EN, we used a powerful approach that combines the precision of ion beam slicing with the imaging capabilities of scanning electron microscopy (SEM), leading to a cutting-edge technique known as focused ion beam (FIB)-SEM tomography. This method enables us to meticulously examine the freshly exposed surfaces of each sliced block of material, capturing a comprehensive set of images that, when combined, form a coherent and accurate volume reconstruction. The utilization of SEM with *Z*-contrast information, specifically employing low-loss backscattered electrons (BSE), was chosen due to the presence of the two distinct chemical phases, HA crystals and Fe-rich pockets, within the Fe-EN. This selection was driven by the need to overcome the challenges associated with weak contrast and the diminutive dimensions of the pockets under investigation. Detailed information on the imaging techniques employed can be found in the Methods, while SI, Figure S8, presents a visual representation of the acquired results.

**Figure 3:**
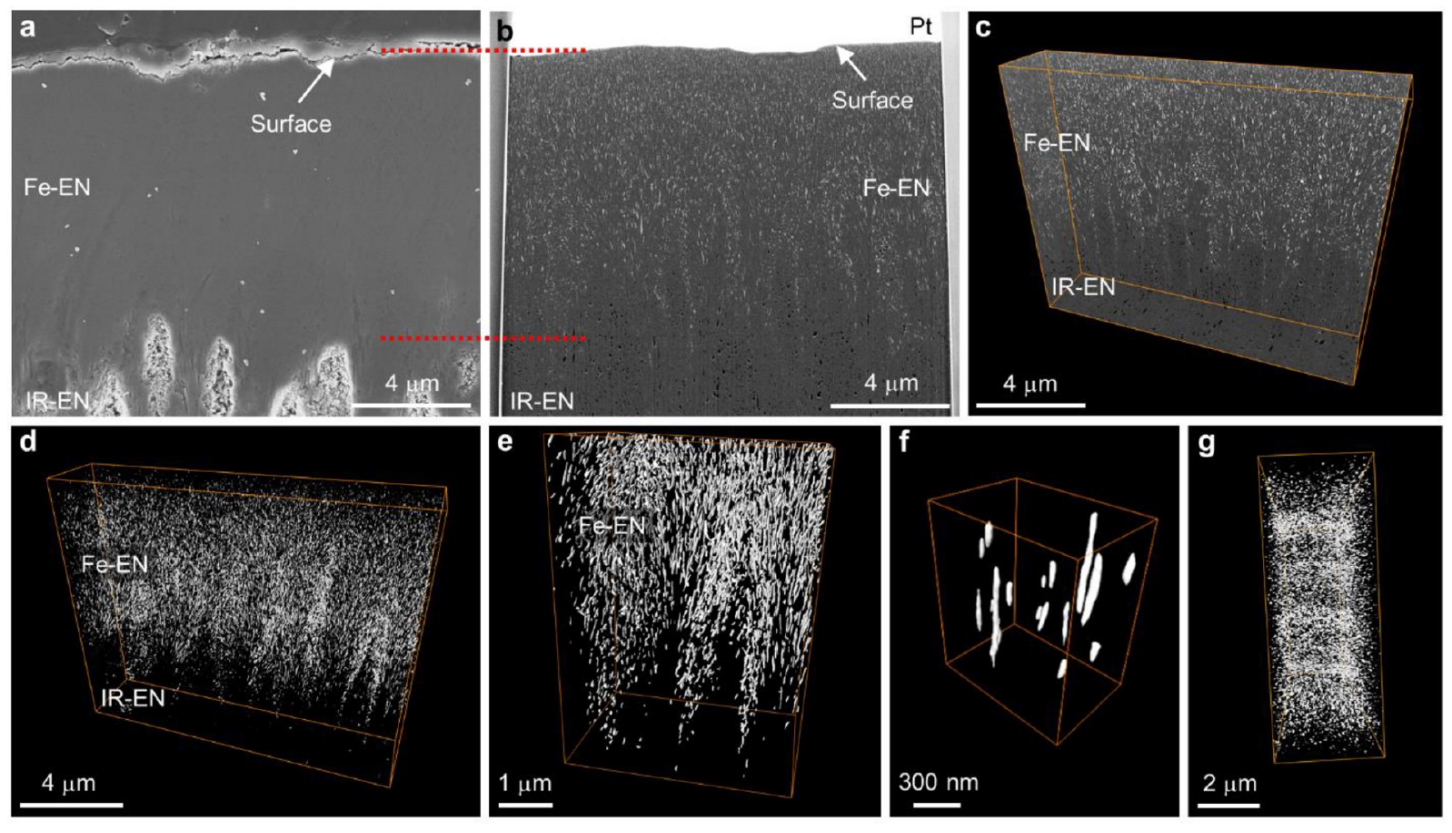
3D spatial arrangement of ferrihydrite-like pockets in Fe-EN. (a) SEM image of an etch-polished cross-section of an incisor showing a region of Fe-EN and a transition to IR-EN. (b) BSE image of a 2D slice cut by FIB from the cross section of an incisor. Images (a) and (b) are from comparable areas. (c) 3D volume combined from aligned 2D serial slice images. (d–g) The 3D reconstruction of filled pockets only within Fe-EN in different orientations; front-view (d–f) at low (d), middle (e), and high (f) magnifications and top-view (g). All data were acquired from the beaver incisor (a–g).

The SEM image of a cross-sectional etch-polished sample (Figure 3a) and a corresponding BSE image of a 2D slice (Figure 3b) extracted from a beaver’s incisor reveal an area encompassing the Fe-EN and its gradual transition toward the inner part of the radial enamel (IR-EN) (SI, Figure S2), providing valuable insights into the enamel’s structural composition. Notably, within the BSE image (Figure 3b), pockets filled with ferrihydrite-like material manifest as distinct bright speckles, while the SEM image of the etch-polished Fe-EN (Figure 3a) does not reveal any recognizable structural features. This disparity arises from the acid-resistant nature of Fe-EN and the specific imaging conditions employed. Additionally, the darker features observed at the bottom of the BSE image (Figure 3b) correspond to empty spaces between HA crystals in the enamel, a structural characteristic that is also evident in the etch-polished regions of the IR-EN. The preferential etching unveils the rod-interrod feature in the IR-EN due to the distinct crystal orientations present. However, the acid-resistant nature of the Fe-EN prevents preferential etching, rendering its enamel structure equivalent throughout (SI, Figure S3e, inset). This direct visual evidence establishes a compelling connection between the pockets filled with ferrihydrite-like material within the Fe-EN and its acid resistant nature.

Through examination and precise alignment of a sequence of 2D serial images, we reconstructed a comprehensive 3D volume of 11.8 × 13.7 × 2.7 μm^3^. This visualization offers insights into the distribution of both filled and empty pockets within the Fe-EN and its transitional region toward the IR-EN (Figure 3c). In the incisors of the beaver, an intriguing network of densely packed, completely filled pockets extends approximately 10 μm deep into the Fe-EN, followed by a gradual decrease in the density of filled pockets. Furthermore, by preparing electron transparent lamellae in a top-view orientation from both the Fe-EN and IR-EN, we were able to directly observe the cross sections of these fascinating, filled, and empty pockets, providing an unobstructed view of their composition and arrangement (SI, Figure S5).

The 3D reconstruction unveils the intricate shape and spatial distribution of the filled pockets within the Fe-EN (Figure 3d–g; SI, Movie S1, Movie S2, and Movie S3), which intriguingly adopt elongated nanosized filaments. Notably, these filaments form a distinct pattern that outlines the rod-interrod structure (Figure 3d–g), providing a visual representation of their arrangement. Leveraging our reconstructed 3D data, we have quantified the volumetric contribution of the ferrihydrite-like material residing within these pockets, revealing a content of 1.67 ± 0.07 vol % in beaver incisors and 1.86 ± 0.07 vol % in coypu incisors, respectively. Furthermore, our detailed analysis exposes a phenomenon where the empty spaces within the enamel structure exhibit a marked preference for occupancy within the rod sheath, as illustrated by the reconstructed 3D network of pockets (Figure 3d,e). This observation provides a deeper understanding of the interplay between the filled and empty spaces within the intricate architecture of the enamel, shedding light on the functional significance of this arrangement.

### Transition Zone and Surface Layer

**Figure 4:**
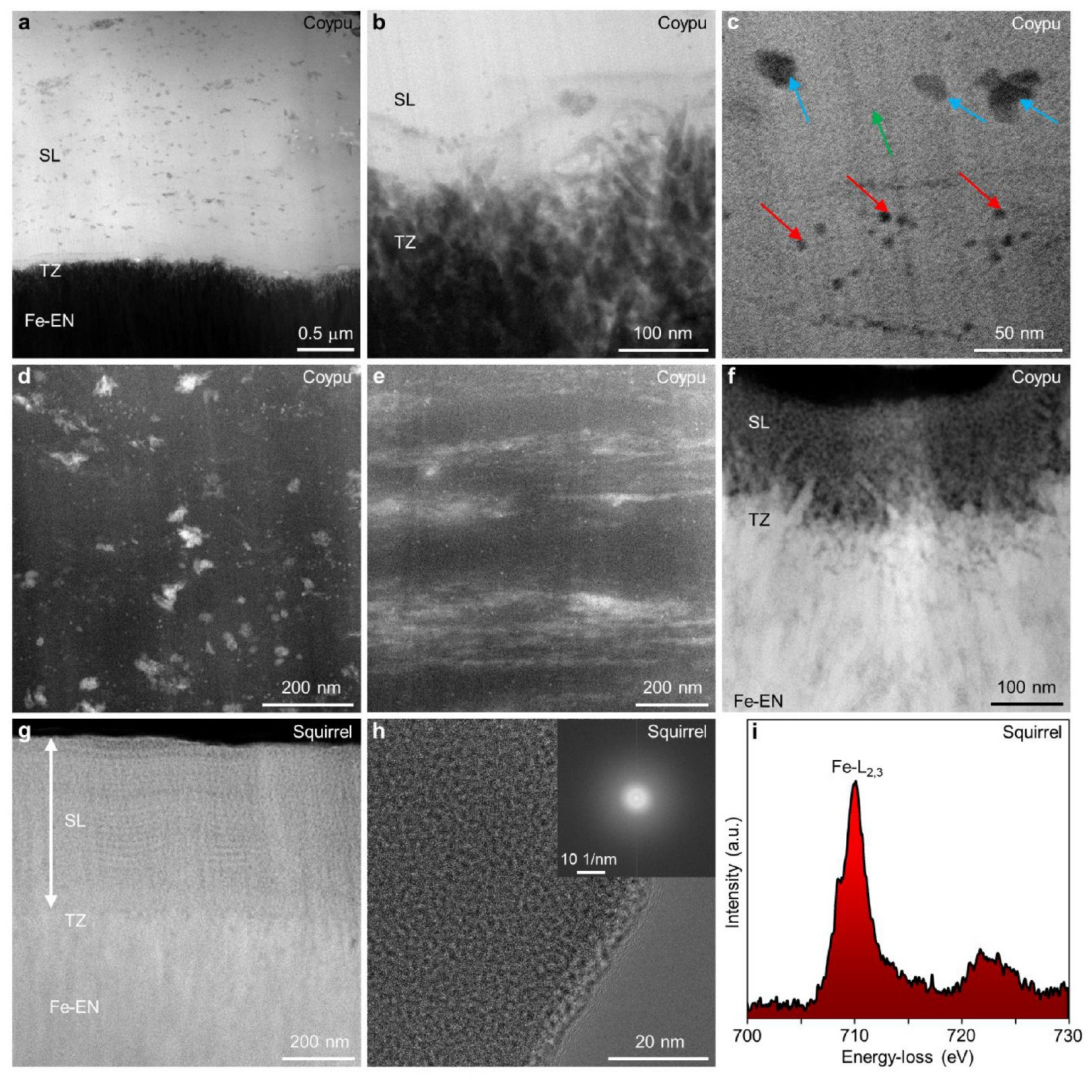
Evolution and characterization of the SL. (a–c) BF-STEM image of cross section showing the interface between the SL and the Fe-EN from the nonerupted part of the incisor at the pigmentation stage. SL consists of organic material with uniformly distributed nanoparticles (a). The relatively irregular enamel surface at the top of the TZ shows presence of HA fragments and smaller HA crystals (a,b). Nanoparticles approach and land on the rough enamel surface (b, see SI, Figure S9b,c). (c) BF-STEM image with marked positions of EDX measurements. Nanoparticles within the SL show the presence of Ca–S–P rich (green and blue arrows) and of Fe–Ca–P–S enriched particles (red arrows) (see SI, Figure S9f,g). (d–f) Evolution of the SL from nonerupted to erupted incisor. SL in the nonerupted incisor shows randomly distributed nanoparticles within the organic matrix (d). In parallel layers aligned nanoparticles within the organic matrix are oriented parallel to the surface of the incisor at the transition area between the nonerupted and erupted parts of the incisor (e). Dense SL in the erupted part of the incisor (f). (g–i) SL covering the incisor in cross-sectional orientation (g) and in plan-view orientation with corresponding SAED pattern that discloses an amorphous state of the SL (h). (i) Fe-L_2,3_ ELNES recorded from the SL shown in (g) showing Fe in predominantly the 3+ oxidation state.

Ameloblasts, anchored to the enamel surface by hemidesmosomes and basal lamina,^3^ play a vital role in enamel biomineralization.^41−44^ Amelotin, an enamel protein expressed during the maturation stage, assumes a strategic role in this process. Its localization in the basal lamina in contact with enamel surface suggests a direct influence on enamel mineralization.^43,45^ Notably, amelotin, particularly its nonphosphorylated peptide fragments, facilitates the phase transformation of acidic amorphous calcium phosphate (ACP) into crystalline HA.^46^ During maturation stage, cyclical pH modulations between weakly acidic and near-neutral conditions induce partial dissolution followed by recrystallization and structural reorganization at the enamel surface.^47,48^ In our investigation across diverse species, we identified common features: a relatively rough enamel surface and a transition zone (TZ) composed of smaller HA fragments blended with Fe-rich material (Figure 4a,b,f,g, and SI, Figure S9a–c,h). The TZ thickness varies, reaching approximately 500 nm in larger animals’ incisors (coypu and beaver). These structural adaptations are crucial for attachment of material formed via ameloblasts through a process of secretion and for the formation of a dense surface layer (SL) parallel to the incisor surface. While the SL has been previously observed in coypu incisors,^8^ its presence in other rodent species has remained unconfirmed.

Figure 4a–f illustrate the progressive evolution of the SL. Our sampling focused on the nonerupted part of the incisor, specifically around the pigmentation stage, allowing us to capture the early stages of SL formation (Figure 4a–d). A low-magnification BF-STEM image (Figure 4a) portrays an organic material infused with nanoparticles firmly attached to the enamel surface. Upon closer examination of the contact region, we observed the precise landing and attachment of nanoparticles onto the rough and disorganized enamel surface, composed of needle-shaped HA crystals and fragments (Figure 4b, and SI, Figure S9a–c). Our analyses reveal two distinct compositional variations within the SL. The round nanoparticles (red arrows, Figure 4c, and SI, Figure S9d–g) are primarily rich in Fe–Ca–P–S, measuring approximately 6 nm in diameter. Conversely, the flake-like nanoparticles and the surrounding matrix exhibit an elevated presence of Ca–S–P (blue and green arrows, Figure 4c, and SI, Figure S9d–g).

Figure 4d–f present the dynamic progression of the SL in the nonerupted stage (Figure 4d), the transition from nonerupted to erupted (Figure 4e), and the erupted stage (Figure 4f) of the incisor. Initially, the secreted nanoparticles are evenly dispersed within an organic matrix (Figure 4d), followed by their reorganization into a layered, uncompressed SL (Figure 4e), ultimately transforming into a dense, intricately intertwined organic–inorganic SL closely integrated with the enamel’s rough surface (Figure 4f).

Interestingly, the SL in squirrel incisors exhibits nearly uniform thickness in side-view samples (Figure 4g). Furthermore, a top-view lamella consisting solely of SL material exhibits a uniform speckled contrast (Figure 4h), as confirmed by the selected area electron diffraction pattern (inset, Figure 4h), indicating the amorphous nature of the material. Through compositional analysis, we characterize the SL covering squirrel incisors as an Fe–Ca phosphate. Notably, analysis of the fine structural details of the Fe-L_2,3_ ELNES reveals the oxidation state of Fe to be predominantly 3+ (Figure 4i).

To gain deeper insight into the chemical composition of the SL material, we conducted a thorough analysis. By examining 338 positions within the SL of all species under investigation, we determined the concentrations of Fe, Ca, and P using experimentally derived EDX k-factors. The data points were then organized based on the decreasing Fe content, as demonstrated in SI, Figure S10. Notably, we observed an intriguing inverse relationship between the compositional profiles of Fe and those of Ca and P (SI, Figure S10), reminiscent of our previous findings concerning the SL of coypu.^8^

The composition of the SL, specifically the Fe–Ca–P ratios, exhibits variability not only between different species but also within the same species and even within individual incisors. This inherent nature of the layer can be attributed to the fact that the SL material originates from various residues secreted by ameloblasts. Consequently, the fluctuating elemental ratios reflect the varying amounts of material retained within ameloblasts following enamel formation including residues of ferritin. The final thickness of the SL is also subject to variability and can be influenced by mechanical abrasion during the gnawing activities. These variations in thickness contribute to local color variations, which are particularly conspicuous in the incisors of larger species, such as beaver and coypu.

### Coloration of Incisors

To unravel the distinctive orange-brown coloration observed in rodent incisors, we conducted a series of experiments to investigate the interplay among the microstructure, chemical composition, and coloration.

**Figure 5:**
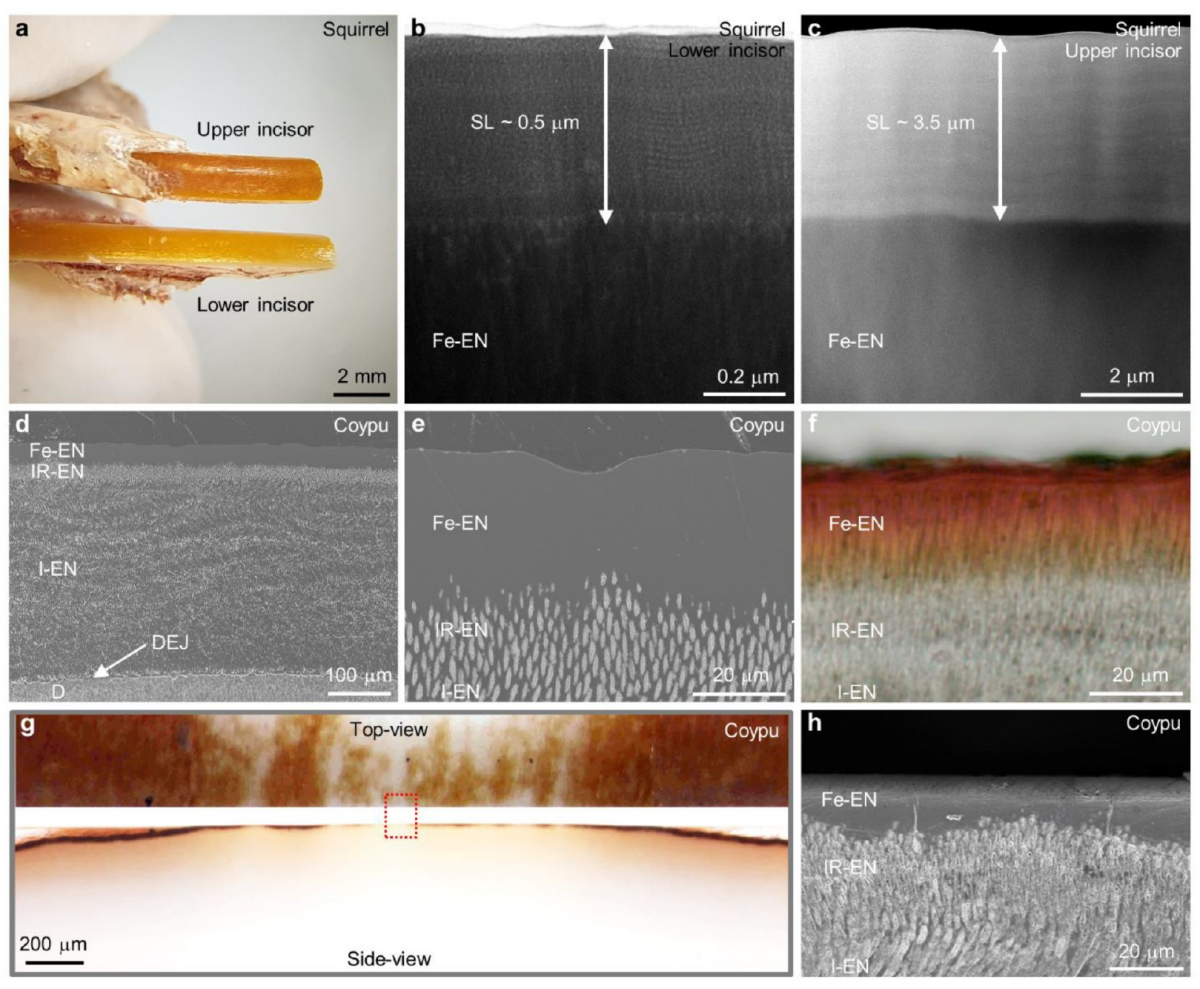
Coloration of rodent incisors. (a) Photograph of upper and lower incisors obtained from a squirrel. The upper incisor appears significantly darker than the lower incisor. (b,c) BF- (b, lower incisors) and HAADF- (c, upper incisors) STEM images of the interface between the SL and the Fe-EN presented in cross-sectional orientation, showing distinctly different thicknesses of the SL. (d) SEM image of an etch-polished cross-section of the incisor of a coypu. (e,f) SEM image of an etch-polished specimen (e) and optical micrograph of a thin polished section (f) of the incisor of a coypu compared at the same magnification. Color transmits from the surface of the incisor toward the interior of the tooth structure only through the thickness of the Fe-EN. (g) Top- and side-views of the incisor of a coypu. The surface of the incisor was polished in a controlled manner to achieve intermediate bright areas. In areas where the top surface appears bright after polishing, reduced or absent color transmission toward the interior of the incisor structure is observed. (h) SEM image of the etch-polished side-view of the sample shown in (g, side-view), corresponding to the area marked with the dotted red square in (g). Although the surface color appears to be light and resembles the color of normal enamel, the SEM image clearly demonstrates the presence of Fe-EN.

First, we observed that the upper incisors of squirrels exhibit a notably darker appearance compared to the lower incisors from the same individual (Figure 5a). Interestingly, in squirrels, we discovered a nearly uniform thickness of the SL within each incisor. Structural and chemical analyses exposed a substantial difference in SL thickness between the upper and lower incisors, with the upper ones approximately seven times thicker than their lower counterparts (Figure 5b,c). Notably, the average chemical composition (Fe/Ca/P at %) did not demonstrate significant differences (upper, 46.3/16.5/37.3; lower, 44.6/18.0/37.5). This clearly suggests a strong correlation between the incisors’ color tone and the thickness of the SL.

Second, a striking effect of color propagation exclusively through the outer radial enamel (Fe-EN) is observed across all studied rodent species (Figure 5d–f, and SI, Figure S6). While the inner part of the radial enamel (IR-EN) displays a similar microstructural organization, only the Fe-EN, with its dense microstructure composed of HA crystals and ferrihydrite-like pockets, serves as the transmission path for the orange-brown color.

Third, we investigated the impact of controlled removal of the orange-brown surface of coypu incisors on color propagation (Figure 5g). By polishing away a thin layer of material from the incisor’s top surface, we obtained intermediate white regions (Figure 5g, top-view). Color propagation was significantly diminished in these areas (Figure 5g, side-view). Although the confined regions appeared similar in color to regular enamel after polishing (Figure 5g), our SEM images of etch-polished sections (side-view) conclusively indicate that all surface regions are part of the Fe-EN (Figure 5h). Furthermore, imaging and analytical TEM measurements confirmed the presence of filled pockets in coypu incisors up to a depth of at least 30 μm from the surface (SI, Figure S11).

The hybrid SL material, comprising an inorganic component with a variable Fe–Ca–P composition intimately blended with an organic matrix, contributes to color generation. Recent studies suggest that aromatic amino acids (AAA), constituents of the organic matrix, may impact the color characteristics of HA.^49^ As AAA are present during enamel development, they likely constitute a part of the residual material that forms the SL.

These findings unveil a completely different understanding of coloration generation in rodent incisors. Accordingly, we propose renaming the previously recognized pigmented enamel to Fe-EN. This structural component is not the primary source of the characteristic color observed on the surface of rodent incisors, as previously believed. Instead, the intense orange-brown coloration predominantly emerges from the SL (and TZ), formed by a combination of organic and inorganic residuals arising from enamel formation processes and pigment release.

Taken together (SI, Figure S12), we investigated the source material contributing to the formation of pockets and the SL, namely, the ferritin nanoparticles, before their secretion from ameloblasts (SI, Figure S12a–c). These nanoparticles exhibit a crystalline nature with low levels of phosphorus, displaying a high Fe/P ratio consistent with mammalian ferritin values. The presence of Fe in its 3+ oxidation state suggests an iron storage mechanism protecting cells from potential toxicity. The Fe-EN comprises elongated HA crystals intertwined with infused ferrihydrite-like material, filling the empty spaces and creating a 3D network of pockets (SI, Figure S12d–f). While the microstructural properties and spatial arrangement of HA crystals in Fe-EN and IR-EN are equivalent, the Fe-EN with its filled pockets offers natural protection against acid attacks. Furthermore, we discovered an additional structural unit on the labial surface of all of the studied rodent species. Following tooth formation processes, remnants excreted from the cells coalesce into the SL, comprising both organic and inorganic material (SI, Figure S12g,h). The incisors’ color tone is directly influenced by the thickness of the SL. Surprisingly, the Fe-EN serves as a path for color transmission rather than being a color-generating component itself (SI, Figure S12i).

## Conclusions

In conclusion, our study unveils insights into the intricately coordinated and regulated biological processes underlying tooth development and architectural construction. While we observed minor microstructural adaptations and slight chemical variations among different species, environmental factors do not appear to exert a dominant influence on tooth development and growth.

The infusion of ferrihydrite-like material into the interstices between elongated hydroxyapatite crystals, along with the formation of the TZ and SL, represents a crucial enhancement of the outer iron-rich radial enamel (Fe-EN). This infusion contributes to improved mechanical properties and enhanced resistance against acid attacks. Although the filled pockets within Fe-EN constitute less than 2% of its volume, they likely play a decisive role in these characteristics. Given the robust and acid-resistant nature of Fe-EN, it does not exhibit coloration. The functional significance of Fe-EN and the understanding of the underlying coloration generation in rodent incisors have far-reaching implications, extending their importance to the development of potentially groundbreaking durable bioinspired and biocompatible dental materials with enhanced properties, inspired by the ingenious designs found in nature. The inclusion of amorphous or nanocrystalline ferrihydrite-like material, as well as other biocompatible iron oxyhydroxides, within everyday dental products could provide exceptional safeguarding for human tooth enamel. Moreover, the subtle infusion of minute quantities of iron oxyhydroxides into engineered synthetic enamel might lead to functionally advanced biomimetic materials. Such advancements have the potential to revolutionize dental care and provide innovative solutions for long-lasting and resilient dental restorations.

## Methods

### Incisor Samples

We analyzed incisors from various rodent species: Eurasian beaver (*Castor fiber*), two animals; feral coypu (*Myocaster coypus*), five animals; alpine marmot (*Marmota marmota*), two animals; grey squirrel (*Sciurus carolinensis*), two animals; European water vole (*Arvicola terrestris*), three animals; rat (*Rattus norvegicus*; from laboratory, Wistar type), three animals; and mouse (*Mus musculus*; from laboratory, CD1 strain), two animals.

A total of 31 ultramicrotomy-prepared samples were obtained from the enamel organ of the investigated species. We also prepared 49 transmission electron microscopy (TEM) sections in a plan-view orientation to examine the pockets in the outer radial enamel (Fe-EN) and inner radial enamel (IR-EN), and 35 TEM sections in a cross-sectional orientation to observe the surface layer (SL), transition zone (TZ), and Fe-EN.

The animals were sourced from different environments: beavers were from the population in the Amberg-Sulzbach region, Germany; coypus were from the non-native population in the Ljubljana swamp, Slovenia; marmots were from the introduced population in Julian Alps, Slovenia; squirrels were from the introduced population in Yorkshire, GB; water voles were from the native population in Notranjska region, Slovenia; rats and mice were from the Centre d’Exploration Fonctionnelle, Cordelier Research Center, Paris, France.

To preserve the tissue integrity, all jaws with teeth were stored at −20 °C throughout the study to prevent decomposition or drying. Teeth were carefully extracted from the jaws, ensuring that the microstructure remained intact. After extraction, the enamel organ was meticulously removed and used for TEM sample preparation through ultramicrotomy. The extracted teeth were then dehydrated using a graded ethanol solution to remove moisture.

### Preparation of Samples for Optical Microscopy, Binocular Observations, Scanning Electron Microscopy (SEM), and Focused Ion Beam (FIB)

Incisors were cut into ∼2–5 mm thick slices in the cross-sectional direction perpendicular to the long axis of the tooth.

#### 1 Optical Microscopy

The slices were carefully glued to a glass plate and stepwise prepared with SiC grinding paper (1200, 2400, and 4000 grit) employed to achieve a refined surface. Subsequently, a polyurethane cloth with a fine alumina suspension containing a 50 nm particle size (Buehler MasterPrep polishing suspension) was utilized to further enhance the smoothness and precision of the samples, resulting in a final thickness of approximately 40–70 μm. Finally, the samples were covered with a thin glass plate for protection.

#### 2 Binocular Observations and Scanning Electron Microscopy (SEM)

The slices were embedded in epoxy and underwent a comprehensive polishing procedure. Initially, SiC grinding paper (1200, 2400, and 4000 grit) was employed for the preparation. Subsequently, a final polishing was meticulously carried out on a polyurethane cloth using an alumina suspension containing 50 nm sized nanoparticles (Buehler MasterPrep polishing suspension).

#### 3 Focused Ion Beam (FIB)

The slices were attached to a Pyrex specimen holder using Crystal Bond thermoplastic wax and polished with SiC grinding paper (1200, 2400, and 4000 grit).

### Preparation of Samples for Experiments with Coloration

Incisors were precisely sectioned into 5 mm thick slices oriented perpendicular to the long axis of the tooth. These slices were then carefully positioned within a square-shaped embedding mold, ensuring the surface side was in close proximity to one edge. Afterward, the samples were prepared with SiC grinding paper (1200, 2400, and 4000 grit) from the side and subsequently from the surface side with SiC grinding paper (4000 grit).

### Transmission Electron Microscopy (TEM) Sample Preparation

The quality of TEM investigations is heavily dependent on meticulous sample preparation, particularly when dealing with biological materials. The transformation from a living hydrated state to a dry state during preparation can introduce significant changes.^50^ To ensure robust and reliable results, we adopted a multifaceted approach to TEM sample preparation.^8,51^ Samples were prepared from the enamel organ and obtained from various positions and orientations on the incisors of all of the investigated rodent species.

It is noteworthy that our TEM samples were deliberately kept free from embedding, staining, and fixing procedures. This strategic decision was made to preserve the pristine chemical composition of the samples and to eliminate any potential sources of contamination or artifacts that could compromise the accuracy of our analytical TEM investigations.

#### a Preparation of Samples from the Enamel Organ by Ultramicrotomy

Samples from the enamel organ, specifically ferritin-filled ameloblasts, were prepared using ultramicrotomy.^50,52,53^ After the incisors were extracted from the jaw, pigmentation stage ameloblasts were identified under a binocular. A small droplet of instant adhesive was carefully applied and allowed to dry in the chosen area. Once dried, the droplet with the attached sample was gently removed using a surgical knife. Another layer of instant adhesive was applied to securely sandwich the sample. The blocks were then trimmed and further refined using an ultramicrotome Leica EM-UC6 (Leica Microsystems, Wetzlar, Germany) equipped with a water-filled diamond knife with a knife angle of 35° (Diatome, Biel, Switzerland). Thin slices with thicknesses ranging from approximately 50 to 300 nm were prepared at room temperature with a knife speed of 1 mm/s and a cleavage angle of 6°. These slices, floating in Ultrapure Millipore water (Billerica, MA, USA), were carefully collected on Cu grids covered with a lacey carbon film.

Importantly, none of the enamel organ samples investigated underwent embedding, staining, or fixing procedures. This deliberate approach was chosen to preserve the samples’ chemical composition and to prevent any potential contamination that could compromise the integrity of our imaging and analytical TEM investigations.

#### b Tripod Polishing Followed by Ar+ Ion-Milling

##### b1 Top-View (Samples Prepared in Plan-View Orientation Parallel to the Surface)

Electron transparent TEM lamellae were prepared from the incisors of various rodent species to facilitate high-resolution imaging and analytical (S)TEM experiments. The incisors were precisely sliced into slabs measuring 1.0–1.5 mm in width, 1.5 mm in length, and 0.5–1.0 mm in thickness, oriented parallel to the tooth surface, using a wire saw (Well, model 3242). These slabs were then affixed to a Pyrex specimen holder by using Crystal Bond thermoplastic wax.

To achieve a planar surface on the inner side of the slab with a target thickness of approximately 50 μm, diamond lapping films (DLFs) of grain sizes of 0.5 and 0.1 μm were employed in a stepwise manner. Polishing was performed using an automated tripod polishing system, the Allied MultiPrep system. Prior to each polishing step, the specimens were carefully inspected by using an optical microscope to ensure optimal progress. Notably, the outer side (surface of the incisor) was intentionally left unpolished.

Subsequently, the samples were securely mounted on 3 mm molybdenum (Mo) half rings using M-Bond 610 epoxy and allowed to dry. For further refinement, all samples underwent precise Ar+ ion-beam thinning in a Gatan Precision Ion polishing system (PIPS II; Gatan, Inc., Pleasanton, CA, USA). The ion milling process involved gradually decreasing the accelerating voltage from 2.5 to 1.0 and 0.3 kV, while maintaining an ion beam angle of 10°. It is worth noting that the samples were cooled during ion milling using liquid nitrogen (L-N_2_) throughout the ion milling process.

##### b2 Side-View (Samples Prepared in Cross-Sectional Orientation Perpendicular to the Surface)

The incisors were cut into slabs measuring 1 mm in width, 2 mm in length, and 1 mm in thickness, perpendicular to the tooth surface, by using a wire saw (Well, model 3242). Subsequently, the slabs were attached to a Pyrex specimen holder by using Crystal Bond thermoplastic wax.

To ensure optimal sample preparation, both sides of the slabs were polished using DLFs with varying grain sizes: 3 μm, 1 μm, and 0.1 μm. The first side was polished to achieve a smooth and planar surface, while the sample thickness was gradually reduced to 300 μm. The slabs were then inverted and reattached to the Pyrex specimen holder, and the second side was polished. This step involved thinning the sample to a thickness of 100 μm and introducing a wedge angle of 1.5–2.0°. Further thinning continued until the desired thickness was 10–15 μm.

To refine the samples further, they were carefully mounted onto 3 mm Mo half rings using adhesive. Subsequently, Ar^+^ ion-beam thinning was performed in a Gatan Precision Ion polishing system (PIPS II; Gatan, Inc., Pleasanton, CA, USA). Throughout the ion milling process, the samples were consistently cooled using L-N_2_. The accelerating voltage was set at 2.2 kV and gradually reduced to 0.35 kV while maintaining an ion beam angle of 8–10°.

#### c Focused Ion Beam (FIB) Preparation of Samples in Top- and Side-View Orientation

TEM samples representing specific areas of rodent incisors were prepared by using the focused ion beam (FIB) technique with the FEI Scios DualBeam system (Thermo Fischer Scientific, Inc.), equipped with a Ga^+^ ion beam source. Cut and polished slices of incisors were fixed to aluminum stubs with the polished side upward for top-view observations. For side-view observations, the incisor slices were fixed perpendicularly to the aluminum stubs, with the natural tooth surface facing upward. To ensure sample stability and protection, a 10 nm thick carbon layer was uniformly deposited using thermal evaporation (Leica EM ACE 600 coater system).

The FIB system was operated at an acceleration voltage of 30 kV. Initially, a protective Pt strap was precisely deposited on the area of interest. Coarse milling commenced with a beam current of 5 nA, which was gradually reduced to 3 and 1 nA. Subsequently, the samples were carefully lifted out using an *in situ* micromanipulator and attached to a half-moon-shaped copper (Cu) ring using Pt. The fixed FIB lamellae were then thinned from both sides at an accelerating voltage of 16 kV and a beam current of 0.25 nA. Further milling was conducted on smaller regions using an accelerating voltage of 8 kV and a beam current of 25 pA, progressively reduced to 5 kV with a beam current of 16 pA. Ultimately, all samples underwent a thorough cleaning step at an accelerating voltage of 2 kV and beam current of 8.9 pA. It is worth noting that our specific requirements necessitated the preparation of extra-large lamellae, measuring 20–40 μm in length. To preserve their mechanical stability, thicker slabs of material were intentionally left between the thinned areas, ensuring structural integrity (see SI, Figure S5h–j).

### Acid Etching

The side-view oriented incisor slices were embedded in epoxy and subjected to a careful polishing procedure. Initially, the samples underwent polishing using SiC grinding paper with grit sizes of 1200, 2400, and 4000 grit, followed by a final polishing step on a polyurethane cloth infused with an alumina suspension containing 50 nm particles (Buehler MasterPrep polishing suspension). Subsequently, a brief treatment with 10% phosphoric acid was employed, after which the samples were thoroughly rinsed with distilled water and gently dried by using compressed air. It is worth mentioning that in orthodontic applications, a 37% phosphoric acid is typically utilized, with varying concentrations and treatment durations reported in the literature to achieve optimal outcomes.^54,55^

### Optical Microscopy

Optical microscopy images were obtained with an optical microscope, an Olympus BH-2. Objective lenses with magnifications of 4×, 10×, 20×, 40×, and 100× (oil) were used.

### Scanning Electron Microscopy (SEM) Imaging

A Zeiss Gemini SEM instrument (Zeiss, DSM 982 Gemini) equipped with an EDX detector and operated at an acceleration voltage of 5 kV was used for imaging of polished and etch-polished incisor surfaces.

### 3D Focused Ion Beam (FIB) Tomography, Data Processing, and 3D Reconstruction

The incisor samples (from beaver and coypu) were carefully prepared for 3D tomography experiments. The samples, with the tooth surface facing upward, were attached to an aluminum stub using conductive silver-filled epoxy (Silver DAG 1415, Agar Scientific) and coated with an 80 nm platinum conductive layer (PECS 682, Gatan, US). A state-of-the-art FIB-SEM instrument (Helios Nanolab 650, Thermo Fisher, USA) was employed for the experimental procedures.

To protect the representative location, a 2 μm Pt layer was deposited on the surface area. The process involved milling a 100 μm wide and 50 μm deep trench in front of the Pt-protected area by using a high current FIB (at an acceleration voltage of 30 kV and a beam current of 21 nA). Subsequently, a U-shaped trench, 25 μm deep, was milled around the volume of interest (at an acceleration voltage of 30 kV and a beam current of 2.5 nA) to prevent material redeposition and shadowing of the imaging signals.

The exposed volume of interest was then serially sectioned and imaged according to the schematic presented in SI, Figure S8. This process was fully automated, utilizing a Python programmed algorithm that incorporated drift correction and auto focusing routines to obtain a series of 2D images with narrow and reproducible spacing between the individual imaging planes. The FIB milling conditions were optimized to prevent beam damage during the serial sectioning operations (acceleration voltage of 30 kV and beam current of 0.23 nA).

SEM images with *Z*-contrast information, utilizing low-loss backscattered electrons (BSE), were acquired at a low-energy electron beam (at an acceleration voltage of 2 kV and a beam current of 200 pA in the UHR-mode), employing an in-column integrated TLD-BSE detector. A 3D data set was acquired using an automated process at a 15 nm slicing step (*z*) with an image pixel size of 7.5 × 7.5 nm^2^ (*x*,*y*). The total probed volumes were 11.8 × 13.7 × 2.7 μm^3^ and 14.2 × 11.5 × 2.9 μm^3^ for coypu and beaver incisor samples, respectively.

The raw 2D image data set was then loaded into Amira 3D processing software (version 2021.2, Thermo Fisher Scientific, US). Adjacent slices within the image sequence were precisely aligned using a least-squares algorithm and registered within 3D data set. To preserve the phase contrast information, the raw BSE images were denoised using nonlocal means (NLM) filtering. Subsequently, a nonsharp masking filter was applied, and the image background was subtracted to enhance the sharpness of the data set details.

The postprocessed data volume accurately reflects the microstructure of the sample, where the brightest, intermediate, and darkest gray levels correspond to pockets, hydroxyapatite, and empty pockets, respectively. The structures of the pockets were highlighted using eigenvalue analysis of the brightest phase based on the Hessian tensor. The distribution of pockets within the entire probed volume was visualized in 3D by using volume rendering techniques.

A subvolume with dimensions of 7.5 × 11.0 × 2.9 μm^3^ was cropped and segmented, assigning it to the individual phases (pockets, incisor matrix, and empty pockets) using a computed 3D watershed transformation algorithm. In the 3D quantification process, the volume fractions of the individual phases were calculated. Finally, the Fe-rich pockets were reconstructed in 3D using triangular approximation and visualized from various perspectives.

### (Scanning) Transmission Electron Microscopy ((S)TEM)

We performed a comprehensive characterization of the samples using advanced transmission electron microscopy techniques. Bright-field (BF) and high-angle annular dark-field (HAADF) scanning transmission electron microscopy (STEM) imaging, combined with energy-dispersive X-ray spectroscopy (EDX) and electron energy-loss spectroscopy (EELS) measurements, were conducted at 60 kV by using a cutting-edge analytical TEM/STEM instrument (JEOL ARM200F, JEOL Co. Ltd.). This instrument features a cold field-emission gun and a DCOR probe Cs-corrector (CEOS Co., Ltd.), ensuring exceptional imaging capabilities. The convergence semi angle of 33.5 mrad resulted in a probe size of 0.1–0.15 nm. HAADF-STEM images were acquired using collection angles of 110–457 mrad to obtain high-resolution details.

Additional investigations, including high-resolution TEM (HR-TEM), electron diffraction, BF- and HAADF-STEM imaging, and EELS measurements, were performed at 60 kV with an advanced TEM/STEM (JEOL ARM200F, JEOL Co. Ltd.) equipped with a cold field-emission gun and a CETCOR image corrector (CEOS Co. Ltd.), enhancing the imaging resolution and accuracy.

EDX spectra and elemental profiles were obtained using a 100 mm^2^ JEOL Centurio SDD-EDX detector and a Thermo Noran System 7 EDX system (Thermo Fisher Scientific Inc.). Box area, line scans, and area maps were acquired to capture the elemental information on the teeth samples. Quantitative EDX analysis utilized experimentally determined *k*-factors obtained from standard specimens under the same experimental conditions as the teeth samples.

EELS spectra and elemental maps (2D spectrum images) were acquired in STEM mode using a postcolumn energy filter with high-speed dual-EELS acquisition capability (Gatan GIF Quantum ERS, Gatan Inc. Pleasanton, USA). An energy dispersion of 0.1 and 0.25 eV/channel resulted in energy resolutions of 0.5–0.6 and 0.75 eV, respectively.

To enhance the quality of the EELS spectra, background subtraction was performed using the power law method.^56^ Moreover, the relative thicknesses (*t*/λ) of the samples were determined using the low-loss EEL spectra and the routine implemented in Digital Micrograph (Gatan), with *t* representing the absolute sample thickness and λ denoting the inelastic mean free path.

### Dose Measurement with AXON Dose

To preserve the integrity of our sensitive materials, we carefully controlled the electron dose to prevent damage. It is crucial to ensure that biominerals and their biocomposites,^57^ such as those containing calcium, are not exposed to electron doses exceeding 10^4^ electrons/nm^2^. To achieve this, we employed the AXON Dose system (Protochips Inc.),^58^ which includes the AXON Dose TEM calibration holder with an integrated Faraday cup at the tip position and the AXON Dose calibration software module. Our samples did not show any visible signs of damage throughout the experiments. During EELS experiments, we took special precautions to preserve the native oxidation state of iron. To achieve this, we conducted our investigations under controlled conditions, where the mean electron dose ranged from 1.15 × 10^4^ to 1.2 × 10^5^ electrons/Å^2^. This ensured that the delicate iron oxidation state remained unaltered, allowing us to accurately analyze it without introducing unwanted changes.

## Data Availability

The data that support the findings of this study are available from the corresponding author upon reasonable request.

## Supporting Information Available

Photographs and 3D scans of rodent incisors from coypu and beaver; SEM images of etch-polished, side-view prepared rodent incisors with marked micro structural components; rodent incisors from macro- to nanoscale showing all constituent elements of incisors; Fe/P ratio measured from of individual ferritin nanoparticles measured by EDX from all investigated rodent species; SEM and HAADF-STEM images showing microstructural differences between Fe-EN and IR-EN; SEM images of etch-polished side-view specimens and optical micrographs of side-view thin sections from all rodent species studied; Orientation of prepared samples of rodent incisors for side- and top-view observations; Graphical description and SEM images of the 3D FIB-SEM tomography technique; STEM images showing evolution of the SL from the nonerupted to the erupted part of the rodent incisor, combined with analytical characterization by EDX spectroscopy; Composition of the SL measured by EDX from numerous positions within the SL and sorted by iron concentration and representative STEM images of the SL covering the beaver incisor; SEM and STEM images of positions in the Fe-EN prepared at different distances from the surface; Evolution of rodent incisors from late pigmentation stage ameloblasts to Fe-EN and SL; Thickness values of total EN, Fe-EN, and R-EN measured from SEM images (PDF)
3D spatial arrangement of ferrihydrite-like pockets in Fe-EN in beaver incisors; the 3D reconstruction of filled ferrihydrite-like pockets is shown at low magnification (MP4)
3D spatial arrangement of ferrihydrite-like pockets in Fe-EN in beaver incisors; the 3D reconstruction of filled ferrihydrite-like pockets is shown at medium magnification (MP4)
3D spatial arrangement of ferrihydrite-like pockets in Fe-EN in beaver incisors; the 3D reconstruction of filled ferrihydrite-like pockets is shown at high magnification (MP4)

## References

[ref1] Chen P.-Y.; McKittrick J.; Meyers M. A. Biological materials: functional adaptations and bioinspired designs. Prog. Mater. Sci. 2012, 57 (8), 1492–1704. 10.1016/j.pmatsci.2012.03.001.

[ref2] Meyers M. A.; Chen P.-Y.; Lin A. Y.-M.; Seki Y. Biological materials: Structure and mechanical properties. Prog. Mater. Sci. 2008, 53 (1), 1–206. 10.1016/j.pmatsci.2007.05.002. 19627786

[ref3] Nanci A. Ten Cate’s Oral Histology. Development, Structure, and Function, 7th ed.; Mosby Elsevier: St. Louis, MO, 2008; 411 pp.

[ref4] Jones F. Teeth and bones: applications of surface science to dental materials and related biomaterials. Surf. Sci. Rep. 2001, 42 (3–5), 75–205. 10.1016/S0167-5729(00)00011-X.

[ref5] Lew A. J.; Beniash E.; Gilbert P. U.; Buehler M. J. Role of the mineral in the self-healing of cracks in human enamel. ACS Nano 2022, 16 (7), 10273–10280. 10.1021/acsnano.1c10407. 35748426

[ref6] Lucas P. W. Dental Functional Morphology: How Teeth Work; Cambridge University Press: Cambridge, UK, 2004; 355 pp.

[ref7] Gordon L. M.; Cohen M. J.; MacRenaris K. W.; Pasteris J. D.; Seda T.; Joester D. Amorphous intergranular phases control the properties of rodent tooth enamel. Science 2015, 347 (6223), 746–750. 10.1126/science.1258950. 25678658

[ref8] Srot V.; Bussmann B.; Salzberger U.; Deuschle J.; Watanabe M.; Pokorny B.; Jelenko Turinek I.; Mark A. F.; van Aken P. A. Magnesium-assisted continuous growth of strongly iron-enriched incisors. ACS Nano 2017, 11 (1), 239–248. 10.1021/acsnano.6b05297. 27936567

[ref9] Risnes S.; Møinichen C.; Septier D.; Goldberg M. Effects of accelerated eruption on the enamel of the rat lower incisor. Advances in Dental Research 1996, 10 (2), 261–269. 10.1177/08959374960100022401. 9206346

[ref10] Pindborg J. The pigmentation of the rat incisor as an index of metabolic disturbances. Oral Surgery, Oral Medicine, Oral Pathology 1953, 6 (6), 780–789. 10.1016/0030-4220(53)90205-9. 13063938

[ref11] Miles A. Pigmented enamel. Proceedings of the Royal Society of Medicine 1963, 56, 918–920. 10.1177/003591576305601032. 14068152 PMC1897598

[ref12] Stein G.; Boyle P. Pigmentation of the enamel of albino rat incisor teeth. Archives of oral biology 1959, 1 (2), 97–105. 10.1016/0003-9969(59)90002-0. 13834100

[ref13] Halse A. Electron microprobe analysis of iron content of incisor enamel in some species of Rodentia. Archives of Oral Biology 1974, 19 (1), 7–11. 10.1016/0003-9969(74)90217-9. 4522933

[ref14] Halse A. Elemental composition of the superficial layer of rat incisor enamel. Calcified Tissue Research 1974, 16, 139–144. 10.1007/BF02008219. 4447893

[ref15] Ungar P. S. Mammal Teeth: Origin, Evolution, And Diversity; Johns Hopkins University Press: Baltimore, MD, 2010; 304 pp.

[ref16] Goldberg M.; Kellermann O.; Dimitrova-Nakov S.; Harichane Y.; Baudry A. Comparative studies between mice molars and incisors are required to draw an overview of enamel structural complexity. Frontiers in Physiology 2014, 5, 359 10.3389/fphys.2014.00359. 25285079 PMC4168675

[ref17] Pham C.-D.; Smith C. E.; Hu Y.; Hu J. C.; Simmer J. P.; Chun Y.-H. P. Endocytosis and enamel formation. Frontiers in physiology 2017, 8, 529 10.3389/fphys.2017.00529. 28824442 PMC5534449

[ref18] Ohshima H.; Maeda T.; Takano Y. Cytochrome oxidase activity in the enamel organ during amelogenesis in rat incisors. Anatomical Record 1998, 252 (4), 519–532. 10.1002/(SICI)1097-0185(199812)252:4<519::AID-AR3>3.3.CO;2-9. 9845203

[ref19] Wen X.; Paine M. L. Iron deposition and ferritin heavy chain (Fth) localization in rodent teeth. BMC research notes 2013, 6, 1 10.1186/1756-0500-6-1. 23281703 PMC3556315

[ref20] Smith C. Cellular and chemical events during enamel maturation. Critical Reviews in Oral Biology & Medicine 1998, 9 (2), 128–161. 10.1177/10454411980090020101. 9603233

[ref21] Zhao H.; Liu S.; Yang X.; Guo L. Role of inorganic amorphous constituents in highly mineralized biomaterials and their imitations. ACS Nano 2022, 16 (11), 17486–17496. 10.1021/acsnano.2c05262. 36255102

[ref22] Harris W. R. Iron Chemistry. In Molecular and Cellular Iron Transport; Templeton D. M., Ed.; CRC Press: New York, 2002; pp 1–40.

[ref23] Linder M. C. Mobilization of stored iron in mammals: a review. Nutrients 2013, 5 (10), 4022–4050. 10.3390/nu5104022. 24152745 PMC3820057

[ref24] Houari S.; Picard E.; Wurtz T.; Vennat E.; Roubier N.; Wu T.; Guerquin-Kern J.-L.; Duttine M.; Thuy T. T.; Berdal A.; et al. Disrupted iron storage in dental fluorosis. Journal of Dental Research 2019, 98, 994–1001. 10.1177/0022034519855650. 31329045

[ref25] Theil E. C.; Matzapetakis M.; Liu X. Ferritins: iron/oxygen biominerals in protein nanocages. JBIC Journal of Biological Inorganic Chemistry 2006, 11 (7), 803–810. 10.1007/s00775-006-0125-6. 16868744

[ref26] Lindley P. F. Iron in biology: a structural viewpoint. Rep. Prog. Phys. 1996, 59 (7), 867 10.1088/0034-4885/59/7/002.

[ref27] Theil E. C. Ferritin: the protein nanocage and iron biomineral in health and in disease. Inorganic chemistry 2013, 52 (21), 12223–12233. 10.1021/ic400484n. 24102308 PMC3882016

[ref28] Theil E. C.; Tosha T.; Behera R. K. Solving biology’s iron chemistry problem with ferritin protein nanocages. Accounts of chemical research 2016, 49 (5), 784–791. 10.1021/ar500469e. 27136423

[ref29] Anderson G. J.; Vulpe C. D. Mammalian iron transport. Cell. Mol. Life Sci. 2009, 66, 3241–3261. 10.1007/s00018-009-0051-1. 19484405 PMC11115736

[ref30] Johnson J. L.; Cannon M.; Watt R. K.; Frankel R. B.; Watt G. D. Forming the phosphate layer in reconstituted horse spleen ferritin and the role of phosphate in promoting core surface redox reactions. Biochemistry 1999, 38 (20), 6706–6713. 10.1021/bi982727u. 10350490

[ref31] Mann S.; Bannister J. V.; Williams R. J. Structure and composition of ferritin cores isolated from human spleen, limpet (Patella vulgata) hemolymph and bacterial (Pseudomonas aeruginosa) cells. Journal of molecular biology 1986, 188 (2), 225–232. 10.1016/0022-2836(86)90307-4. 3088283

[ref32] Mann S. The role of inorganic phosphate in iron oxide biomineralization. In Origin, Evolution, And Modern Aspects of Biomineralization in Plants and Animals, International Symposium on Biomineralization; Crick R. E., Ed.; Plenum Press: Arlington, TX, 1989; pp 273–288.

[ref33] Kallenbach E. Fine structure of rat incisor enamel organ during late pigmentation and regression stages. Journal of ultrastructure research 1970, 30 (1–2), 38–63. 10.1016/S0022-5320(70)90063-8. 5411814

[ref34] Halse A.; Selvig K. Incorporation of iron in rat incisor enamel. European Journal of Oral Sciences 1974, 82 (1), 47–56. 10.1111/j.1600-0722.1974.tb01900.x. 4522964

[ref35] Madsen M. B.; Mørup S.; Koch C. J.; Lindemann G. A study of the sump beaver’s dental enamel. Hyperfine Interact. 1986, 29, 1431–1434. 10.1007/BF02399503.

[ref36] Stifler C. A.; Jakes J. E.; North J. D.; Green D. R.; Weaver J. C.; Gilbert P. U. Crystal misorientation correlates with hardness in tooth enamels. Acta Biomaterialia 2021, 120, 124–134. 10.1016/j.actbio.2020.07.037. 32711081

[ref37] Pan Y.-H.; Sader K.; Powell J. J.; Bleloch A.; Gass M.; Trinick J.; Warley A.; Li A.; Brydson R.; Brown A. 3D morphology of the human hepatic ferritin mineral core: New evidence for a subunit structure revealed by single particle analysis of HAADF-STEM images. J. Struct. Biol. 2009, 166 (1), 22–31. 10.1016/j.jsb.2008.12.001. 19116170 PMC2832756

[ref38] Quintana C.; Cowley J.; Marhic C. Electron nanodiffraction and high-resolution electron microscopy studies of the structure and composition of physiological and pathological ferritin. J. Struct. Biol. 2004, 147 (2), 166–178. 10.1016/j.jsb.2004.03.001. 15193645

[ref39] Desilva D.; Guo J.-H.; Aust S. D. Relationship between iron and phosphate in mammalian ferritins. Archives of biochemistry and biophysics 1993, 303 (2), 451–455. 10.1006/abbi.1993.1308. 8512327

[ref40] Muto T.; Miyoshi K.; Horiguchi T.; Noma T. Dissection of morphological and metabolic differentiation of ameloblasts via ectopic SP6 expression. Journal of Medical Investigation 2012, 59, 59–68. 10.2152/jmi.59.59. 22449994

[ref41] Iwasaki K.; Bajenova E.; Somogyi-Ganss E.; Miller M.; Nguyen V.; Nourkeyhani H.; Gao Y.; Wendel M.; Ganss B. Amelotin—a novel secreted, ameloblast-specific protein. Journal of dental research 2005, 84 (12), 1127–1132. 10.1177/154405910508401207. 16304441

[ref42] Somogyi-Ganss E.; Nakayama Y.; Iwasaki K.; Nakano Y.; Stolf D.; McKee M. D.; Ganss B. Comparative temporospatial expression profiling of murine amelotin protein during amelogenesis. Cells Tissues Organs 2012, 195 (6), 535–549. 10.1159/000329255. 21912076

[ref43] Abbarin N.; San Miguel S.; Holcroft J.; Iwasaki K.; Ganss B. The enamel protein amelotin is a promoter of hydroxyapatite mineralization. Journal of Bone and Mineral Research 2015, 30 (5), 775–785. 10.1002/jbmr.2411. 25407797

[ref44] Nakayama Y.; Holcroft J.; Ganss B. Enamel hypomineralization and structural defects in amelotin-deficient mice. Journal of dental research 2015, 94 (5), 697–705. 10.1177/0022034514566214. 25715379

[ref45] Dos Santos Neves J.; Wazen R. M.; Kuroda S.; Francis Zalzal S.; Moffatt P.; Nanci A. Odontogenic ameloblast-associated and amelotin are novel basal lamina components. Histochemistry and cell biology 2012, 137, 329–338. 10.1007/s00418-011-0901-4. 22231912

[ref46] Zhang J.; Wang L.; Zhang W.; Putnis C. V. Phosphorylated/nonphosphorylated motifs in amelotin turn off/on the acidic amorphous calcium phosphate-to-apatite phase transformation. Langmuir 2020, 36 (8), 2102–2109. 10.1021/acs.langmuir.9b02735. 32036670

[ref47] Smith C.; Issid M.; Margolis H.; Moreno E. Developmental changes in the pH of enamel fluid and its effects on matrix-resident proteinases. Advances in dental research 1996, 10 (2), 159–169. 10.1177/08959374960100020701. 9206332

[ref48] Lacruz R. S.; Nanci A.; Kurtz I.; Wright J. T.; Paine M. L. Regulation of pH during amelogenesis. Calcified tissue international 2010, 86, 91–103. 10.1007/s00223-009-9326-7. 20016979 PMC2809306

[ref49] Guo Y.; Yang X.; Feng X.; Sa Y.; Wang M.; Li P.; Jiang T. New insights into effects of aromatic amino acids on hydroxyapatite. Journal of Dental Research 2018, 97 (4), 402–408. 10.1177/0022034517741274. 29130776

[ref50] Hagler H. K. Ultramicrotomy for biological electron microscopy. Electron microscopy: methods and protocols 2007, 369, 67–96. 10.1007/978-1-59745-294-6_5. 17656747

[ref51] Srot V.; Bussmann B.; Salzberger U.; Koch C. T.; van Aken P. A. Linking microstructure and nanochemistry in human dental tissues. Microscopy and Microanalysis 2012, 18 (3), 509–523. 10.1017/S1431927612000116. 22494533

[ref52] Reid N. Ultramicrotomy; Practical Methods in Electron Microscopy; Elsevier Science Ltd, 1975; Vol. 3, pp 215–353.

[ref53] Michler G. H.; Lebek W. Ultramikrotomie in der Materialforschung; Hanser: München, 2004; 241 pp.

[ref54] Silverstone L.; Saxton C.; Dogon I. L.; Fejerskov O. Variation in the pattern of acid etching of human dental enamel examined by scanning electron microscopy. Caries research 1975, 9 (5), 373–387. 10.1159/000260179. 1055640

[ref55] Legler L.; Retief D.; Bradley E. Effects of phosphoric acid concentration and etch duration on enamel depth of etch: an in vitro study. American Journal of Orthodontics and Dentofacial Orthopedics 1990, 98 (2), 154–160. 10.1016/0889-5406(90)70009-2. 2198801

[ref56] Egerton R. F. Electron Energy-Loss Spectroscopy in the Electron Microscope, 3rd ed.; Springer Science & Business Media: New York, 2011; 491 pp.

[ref57] Kłosowski M. M.; Friederichs R. J.; Nichol R.; Antolin N.; Carzaniga R.; Windl W.; Best S. M.; Shefelbine S. J.; McComb D. W.; Porter A. E. Probing carbonate in bone forming minerals on the nanometre scale. Acta Biomaterialia 2015, 20, 129–139. 10.1016/j.actbio.2015.03.039. 25848725

[ref58] Damiano J.; Walden S.; Franks A.; Marusak K.; Larson B.; Coy M.; Nackashi D. AXON Dose: A Solution for Measuring and Managing Electron Dose in the TEM. Microscopy Today 2022, 30 (4), 22–25. 10.1017/S1551929522000840.

