# Supplementary material for: Ingenious Architecture and Coloration Generation in Enamel of Rodent Teeth
Source: ACS Nano. 2024 Apr 17;18(17):11270–83. doi: 10.1021/acsnano.4c00578 (PMC11064225; doi:10.1021/acsnano.4c00578)
Supplement: Supplementary file 1 — nn4c00578_si_001.pdf [file nn4c00578_si_001.pdf]

Supporting Information

# Ingenious Architecture and Coloration Generation in Enamel of Rodent Teeth

*Vesna Srot[1]*, Sophia Houari[2,3,†], Gregor Kapun[4,5,†], Birgit Bussmann[1,‡], Felicitas Predel[1,‡], Boštjan Pokorny[6,7], Elena Bužan[8,6], Ute Salzberger[1], Bernhard Fenk[1], Marion Kelsch[1], and Peter A. van Aken[1]*

1. Max Planck Institute for Solid State Research, Stuttgart, 70569, Germany.
2. Unité de Formation et de Recherche d'Odontologie, Université Paris Cité, Paris, 75006, France.
3. UR2496, Biomedical research in Odontology, Université Paris Cité, Montrouge, 92120, France.
4. National Institute of Chemistry, Ljubljana, 1000, Slovenia.
5. Centre of Excellence on Nanoscience and Nanotechnology - Nanocenter, Ljubljana, 1000, Slovenia.
6. Faculty of Environmental Protection, Velenje, 3320, Slovenia.
7. Slovenian Forestry Institute, Ljubljana, 1000, Slovenia.
8. Faculty of Mathematics, Natural Sciences and Information Technologies, University of Primorska, Koper, 6000, Slovenia.

†, ‡ These authors contributed equally.

*Corresponding author:

Vesna Srot (V.Srot@fkf.mpg.de)

**Supporting Information:**

- **Supporting Figures (1-12)**
- **Supporting Table (1)**
- **Supporting Movies (1-3)**

## Supporting Figures

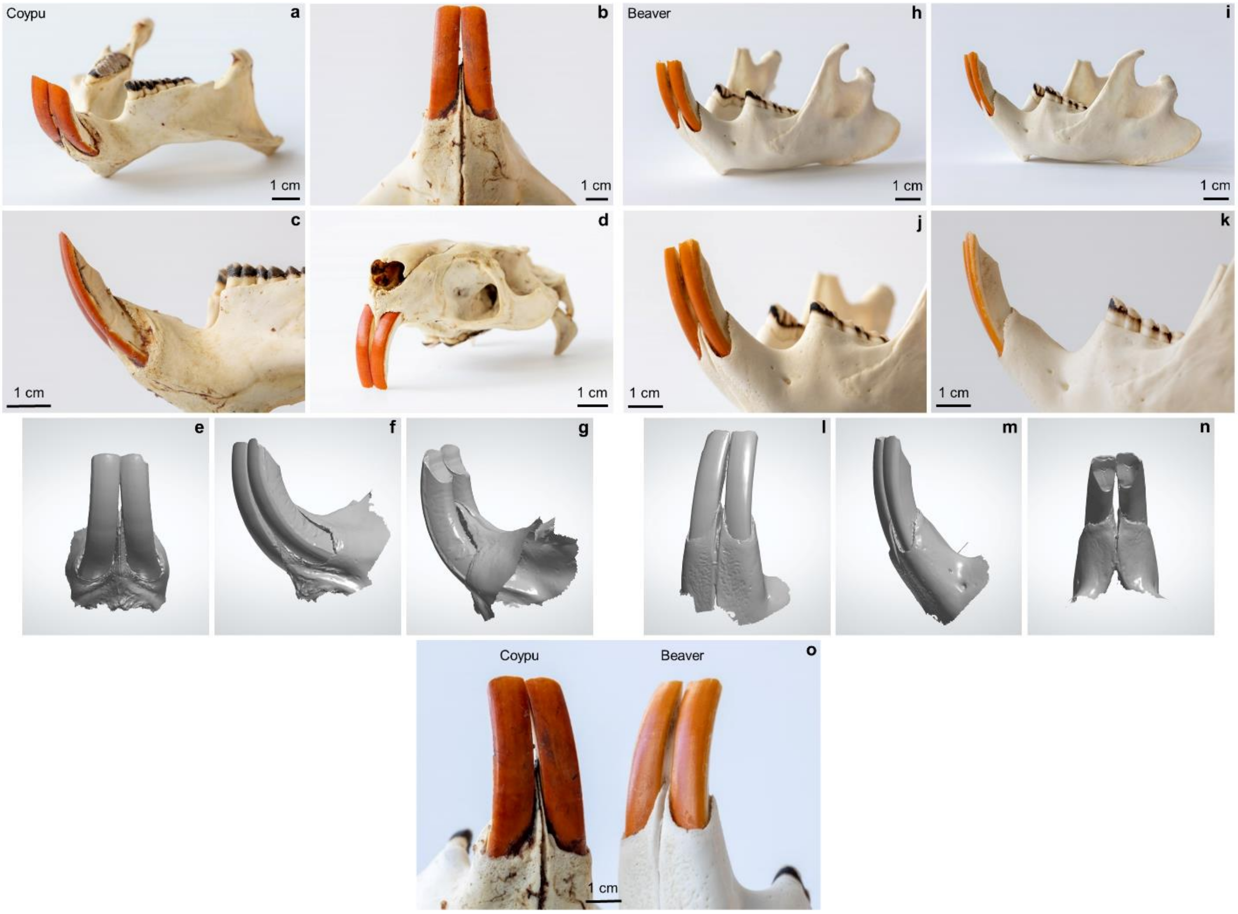


**Figure S1.** Photographs and 3D scans of rodent incisors. (a-g) Photographs of the lower (a-c) and upper (d) jaws of the coypu with corresponding 3D scans of the lower incisors (erupted part) (e-g). (h-n) Photographs of beaver lower jaws (h-k) with corresponding 3D scans (l-n). (o) Photograph of lower incisors (erupted part) of coypu and beaver showing representative difference in color.

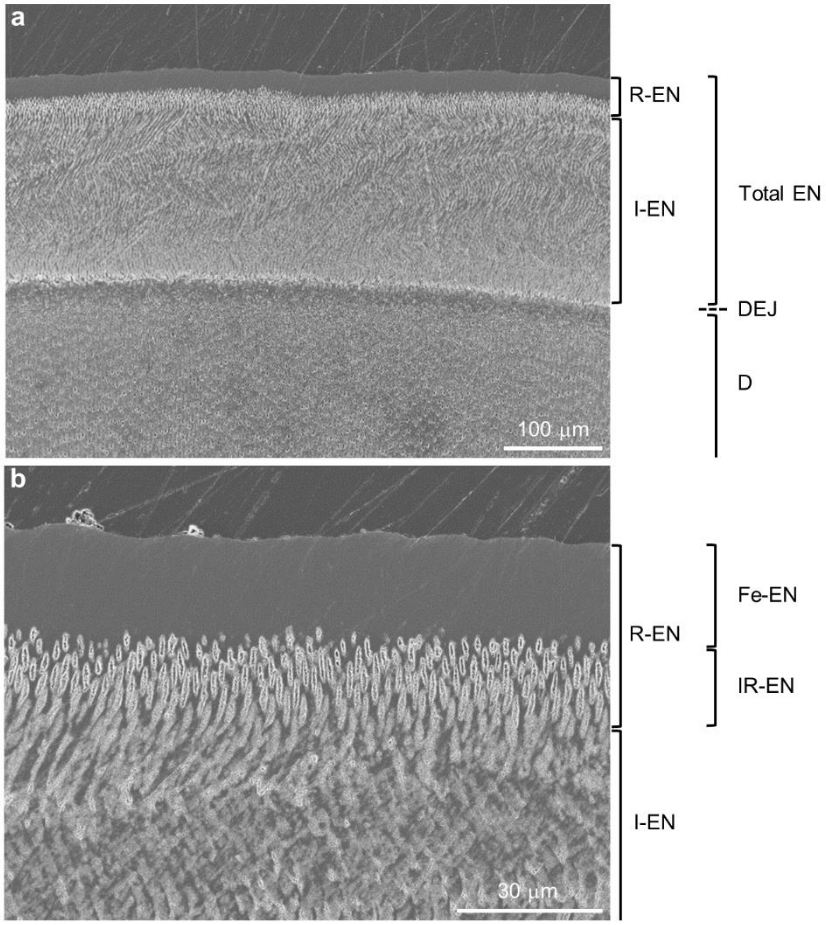


**Figure S2.** Description of structural components of rodent incisors. (a-b) SEM images of an etch-polished side-view prepared incisor of coypu with marked positions of different structural components. Total EN: total enamel, DEJ: dentin-enamel junction, D: dentin, R-EN: radial enamel, I-EN: inner enamel, Fe-EN: Fe-rich enamel, IR-EN: inner radial enamel.

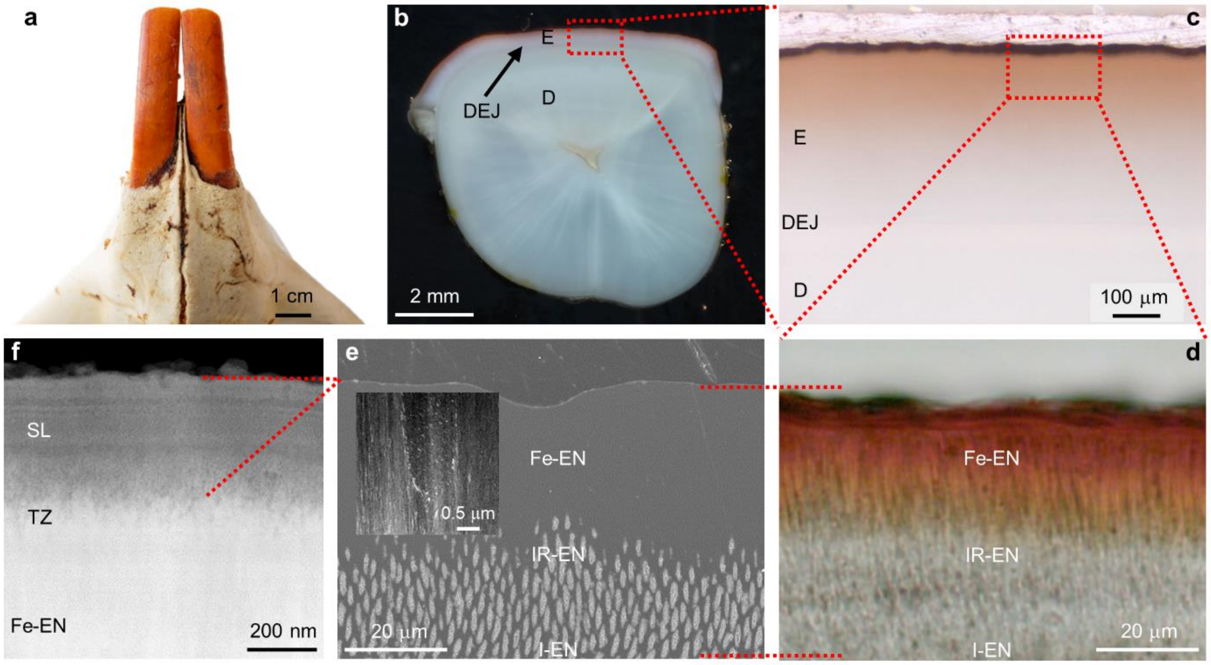


**Figure S3.** Incisors of rodents from macro- to nanoscale. (a) Lower incisors of coypu inside the jaw. (b) Binocular image of incisor cross section showing E (enamel), DEJ (dentin-enamel junction) and D (dentin). (c) Binocular image of magnified side-view area showing E, DEJ and D. (d) Optical micrograph of magnified side-view area showing Fe-EN, IR-EN and I-EN. (e) SEM image of etch-polished side-view prepared specimen showing Fe-EN, IR-EN and I-EN. BF-STEM image showing rod-interrod structure in Fe-EN (inset). (d) and (e) are shown at the same magnification for direct comparison. Color is transmitted from the surface only through the thickness of Fe-EN. (f) HAADF-STEM image of SL covering the incisor. All images (a-f) are from coypu incisors.

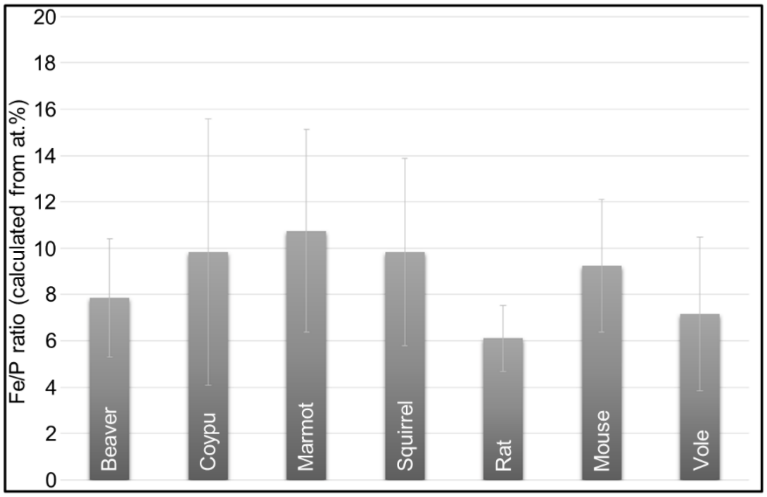


**Figure S4.** Fe/P ratio measured from single ferritin nanoparticles. For each investigated rodent species, 13-20 single ferritin nanoparticles from ameloblasts at the pigmentation stage were measured by EDX. Quantification was performed using experimentally determined k-factors and Fe/P ratios were determined using Fe-K and P-K lines. The values are in good agreement with mammalian ferritin. The diagram shows the mean values of all measurements together with the corresponding standard deviations. Please note that our samples were not chemically treated, fixed or stained in order to preserve their native state and composition. In addition, they have not been extracted from native environments and consequently containing small amounts of P (as determined by EDX). Therefore, the actual Fe/P ratios are even higher than presented here.

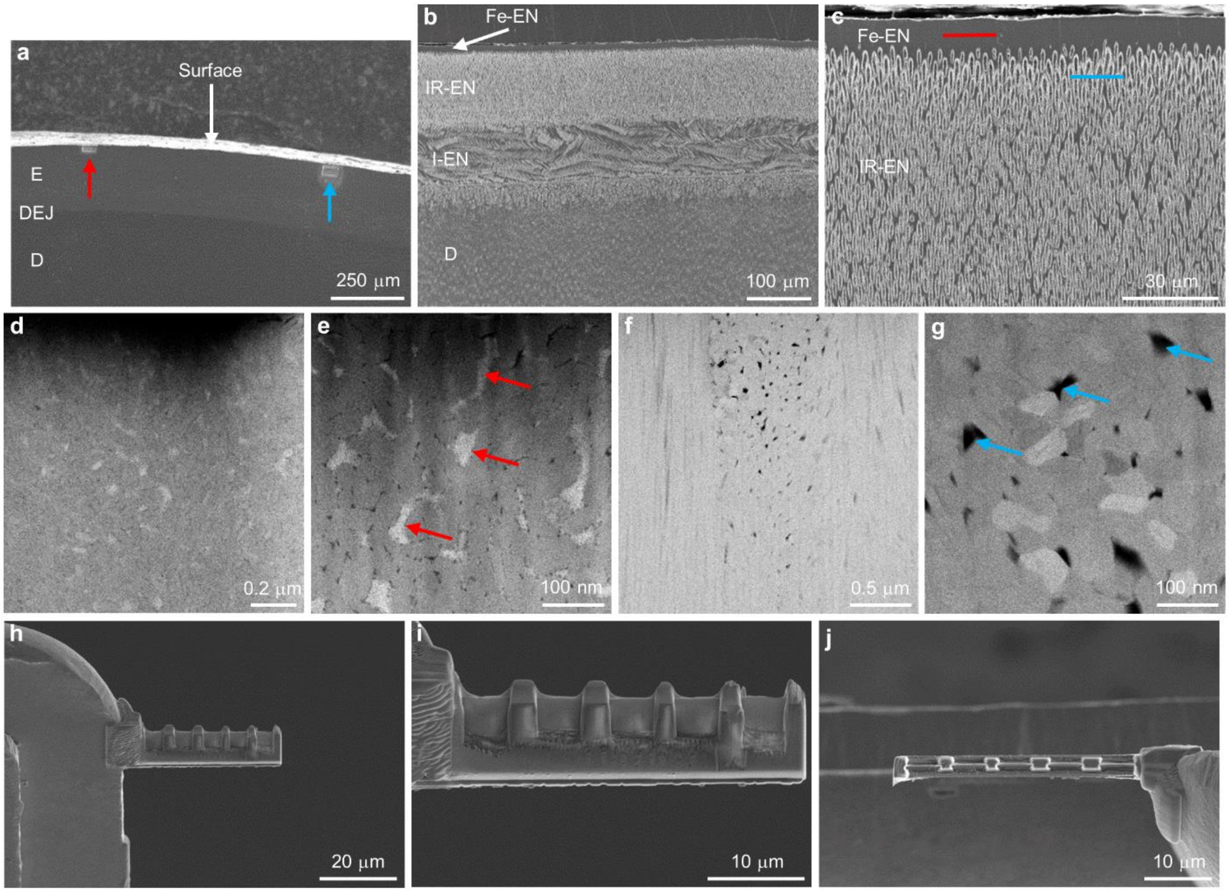


**Figure S5.** Microstructure of Fe-EN vs. IR-EN. (a) SEM image of the side view of an incisor with positions (red and blue arrows) of areas used for site-specific thin sections in top-view orientation. (b) Low-magnification SEM image of etch-polished side-view specimen showing Fe-EN, IR-EN, I-EN and D. (c) Higher-magnification SEM image of etch-polished side-view specimen with marked positions from which specimen of Fe-EN (red) and IR-EN (blue) were prepared. (d-e) HAADF-STEM images of the top-view prepared Fe-EN specimen (red arrow in a and red line in c) with marked pockets that are filled with ferrihydrite-like material (red arrows). (f-g) HAADF-STEM images of the top view IR-EN specimen (blue arrow in a and blue line in c) showing empty pockets between HA crystals. (h-j) Extra-large FIB lamella prepared from Fe-EN. Several areas have been thinned to electron transparency. To achieve mechanical stability of the prepared lamella, we left thicker bridges between the thinned areas.

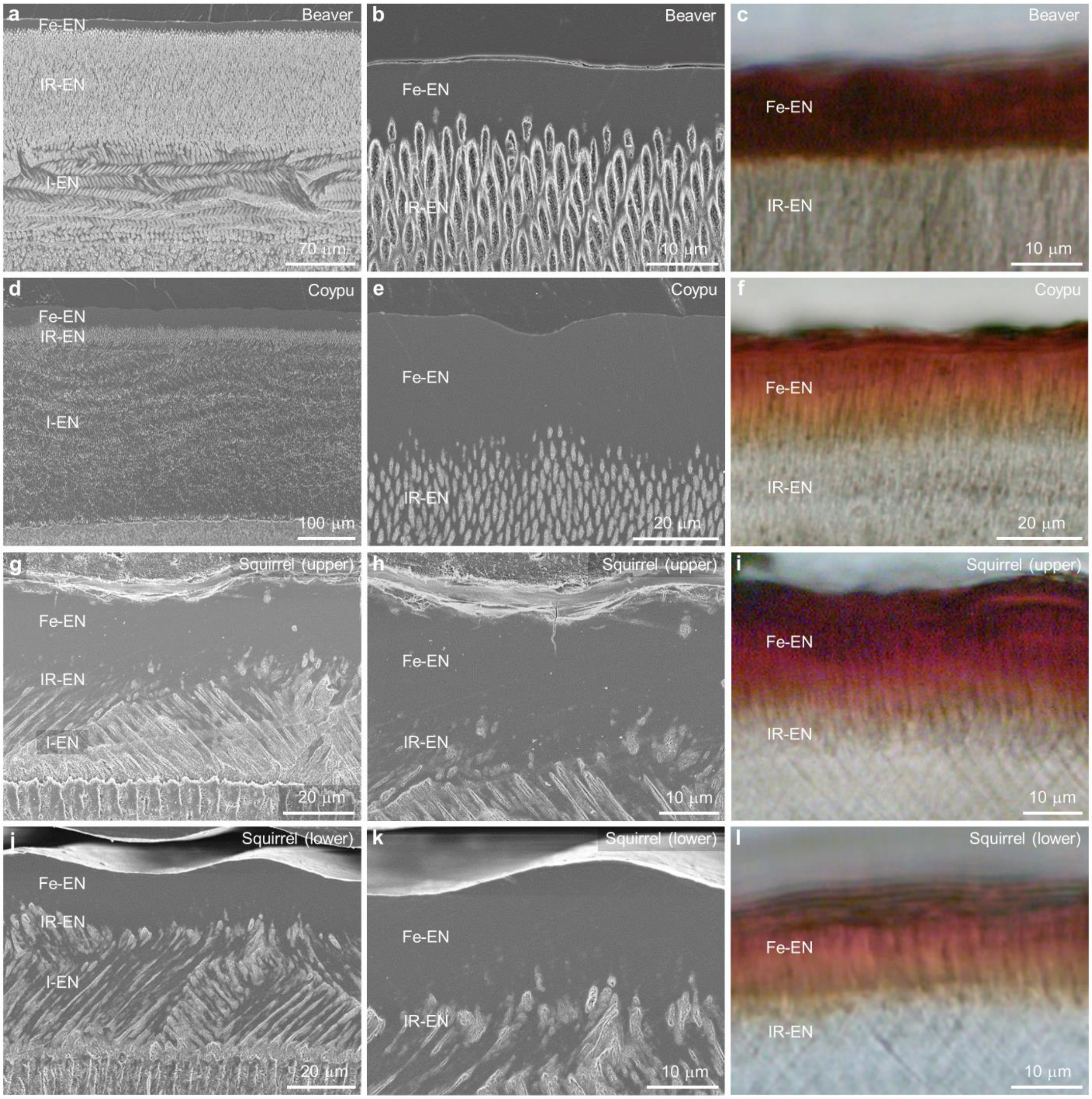
a
Beaver
Fe-EN
IR-EN
I-EN
70 μm
b
Beaver
Fe-EN
IR-EN
10 μm
c
Beaver
Fe-EN
IR-EN
10 μm
d
Coypu
Fe-EN
IR-EN
I-EN
100 μm
e
Coypu
Fe-EN
IR-EN
20 μm
f
Coypu
Fe-EN
IR-EN
20 μm
g
Squirrel (upper)
Fe-EN
IR-EN
I-EN
20 μm
h
Squirrel (upper)
Fe-EN
IR-EN
10 μm
i
Squirrel (upper)
Fe-EN
IR-EN
10 μm
j
Squirrel (lower)
Fe-EN
IR-EN
I-EN
20 μm
k
Squirrel (lower)
Fe-EN
IR-EN
10 μm
l
Squirrel (lower)
Fe-EN
IR-EN
10 μm

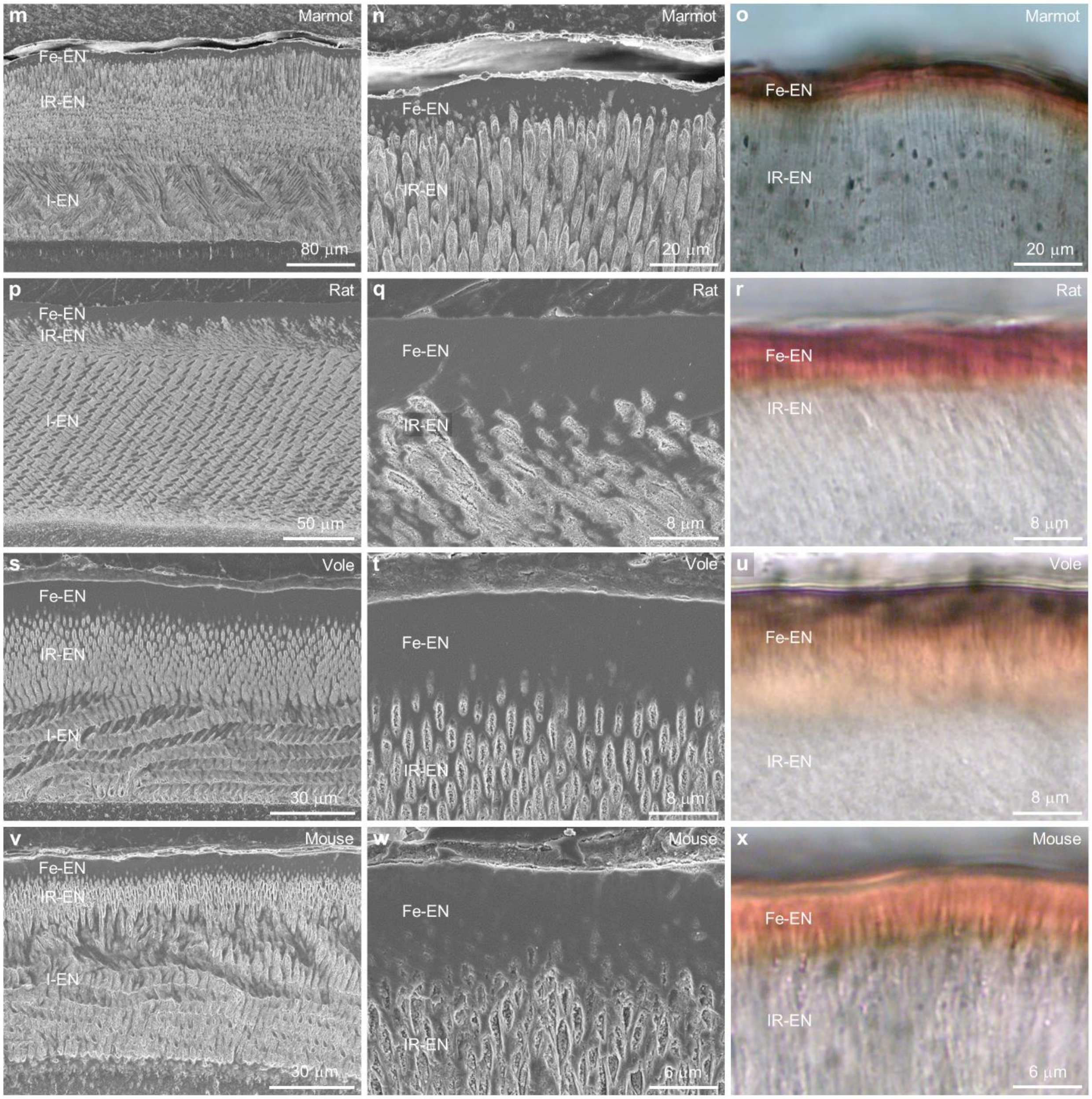


**Figure S6.** SEM images of etch-polished side-view specimens of rodent incisors and optical micrographs of side-view thin sections. SEM images of etched specimens and optical micrographs of side-view thin sections from all rodent species studied (beaver a-c, coypu d-f, squirrel g-l, marmot m-o, rat p-r, vole s-u, mouse v-x). Low-magnification SEM images of etch-polished specimens – left column. SEM images of etch-polished samples (middle column) and optical micrographs of thin sections (right column) are shown at the same magnification for direct

comparison. The color radiates from the surface only through the acid-resistant Fe-EN for all species studied. See Supporting Table 1.

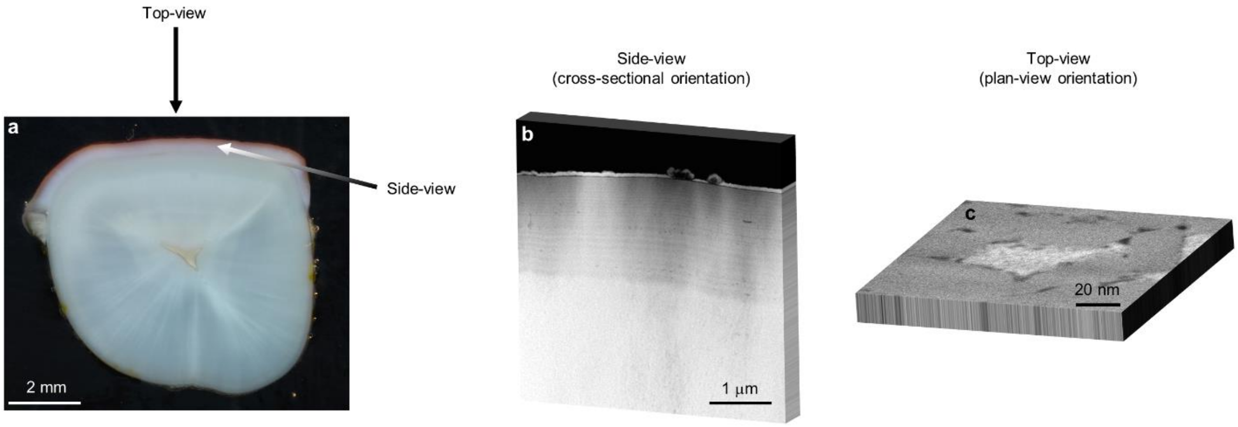


**Figure S7.** Orientation of prepared samples. (a) Slice cut from a coypu incisor with viewing directions marked. For observation of the incisor (side-view), the specimen is prepared in cross-sectional orientation. For observation from above (top-view), the specimen is prepared parallel to the surface of the incisor in plan-view orientation. (b-c) Corresponding HAADF-STEM images of TEM specimens prepared in side-view (b) and top-view (c) orientation.

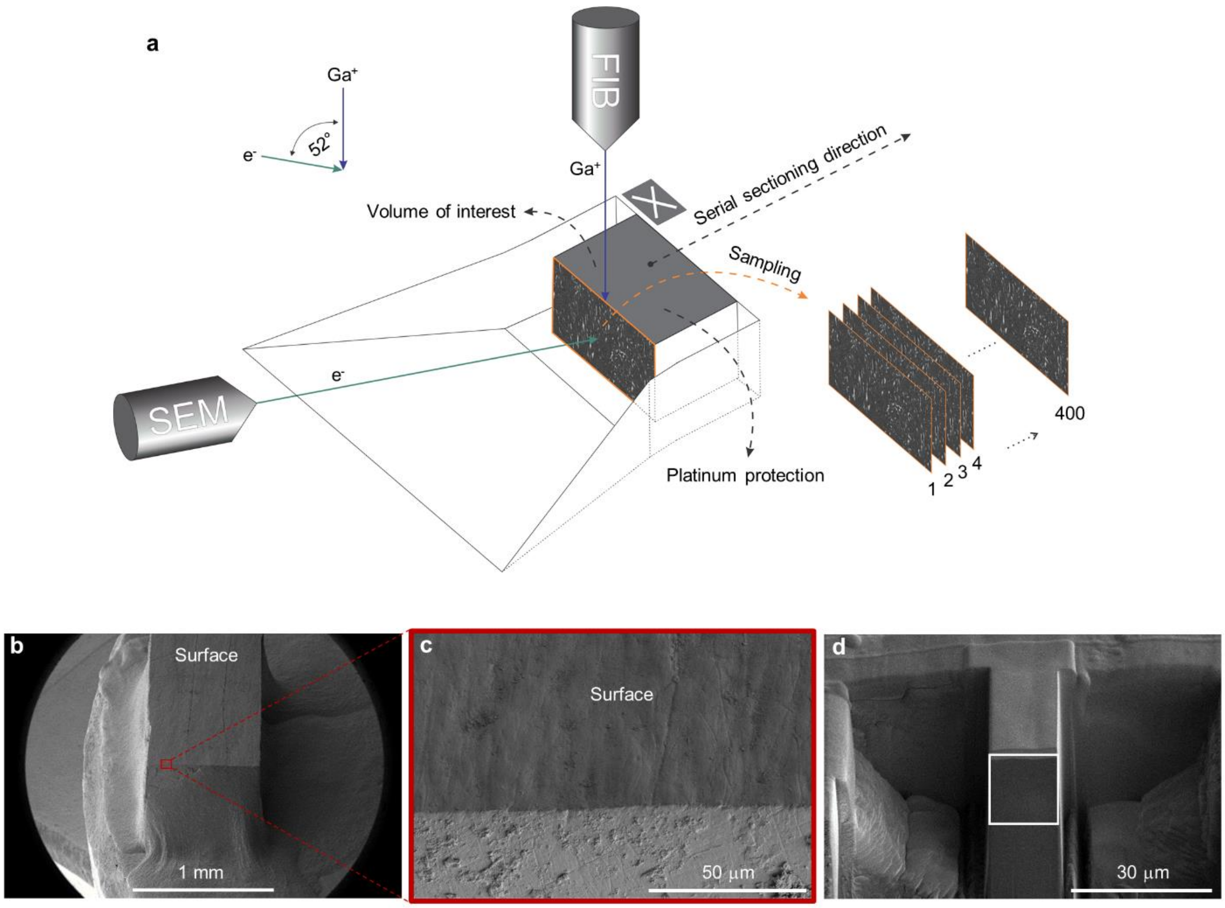


**Figure S8.** FIB sample preparation, 3D data acquisition and reconstruction by slice and view technique. (a) Sketch of the experimental setup used for 3D slice and view experiments. (b-d) SEM images of the sample area used for FIB slicing.

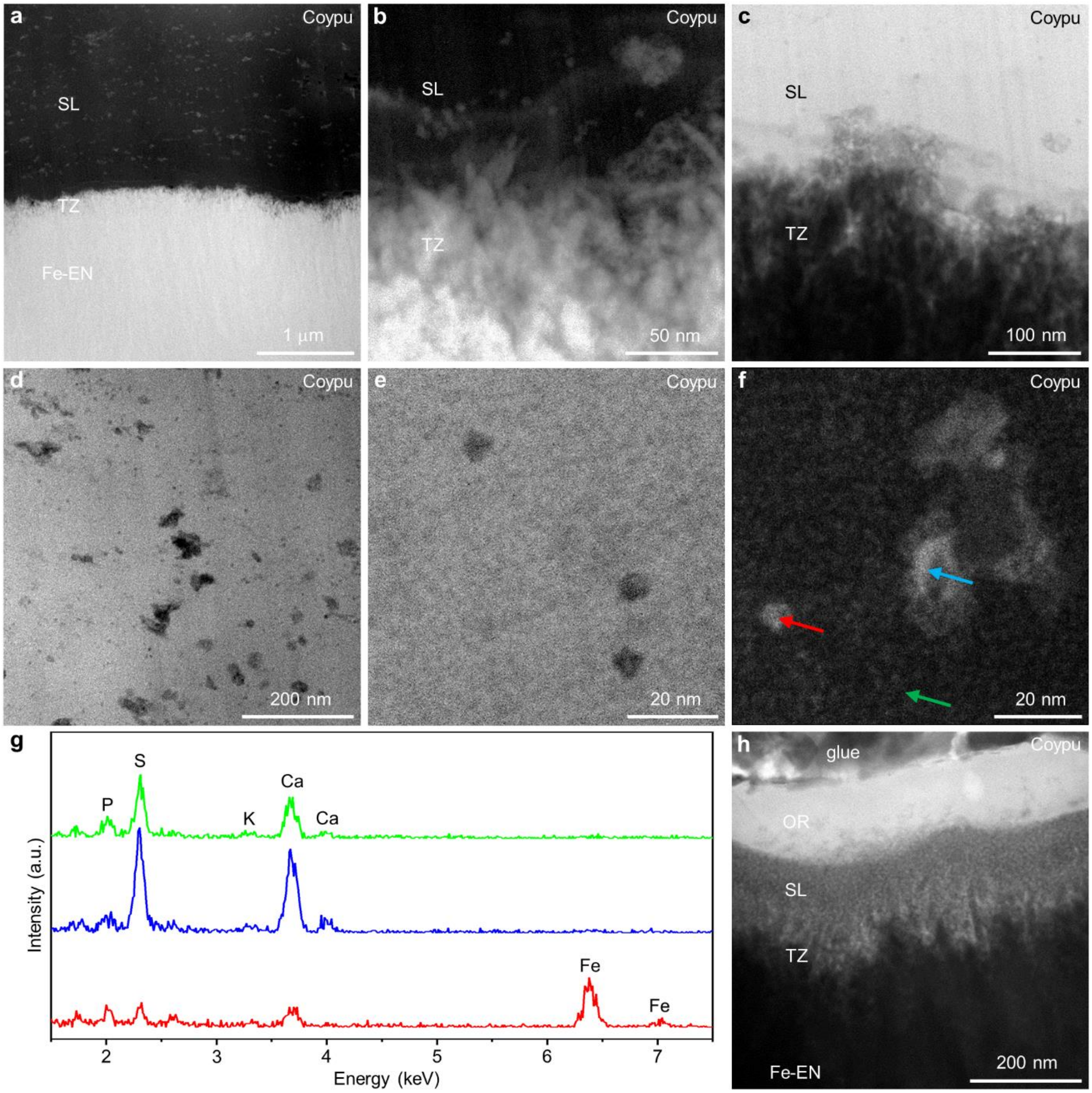


**Figure S9.** Development of the SL. (a-c) HAADF-STEM images (a and b) and BF-STEM images (c) of the interface between the SL and the Fe-EN on the non-erupted part of the incisor at the pigmentation stage (see Fig. 4a, b). Nanoparticles within the organic material of the SL are moving towards (b) and some are already attached to the rough enamel surface (c). (d-e) BF-STEM images of the SL (presented in a-c) showing flake-like nanoparticles with sizes up to 20 nm (d) and smaller round nanoparticles with sizes of around 6 nm (e). The organic material appears to be saturated

with even smaller (about 1 nm or less) particles (e). (f-g) HAADF-STEM image (f) and EDX spectra (g) of flake-like nanoparticles (blue arrow) and the smallest nanoparticles (green arrow) show Ca-S-P enrichment. The round nanoparticles (red arrow) are enriched in Fe-Ca-P-S. (h) BF-STEM image of the SL attached to the enamel surface of the erupted part of the incisor.

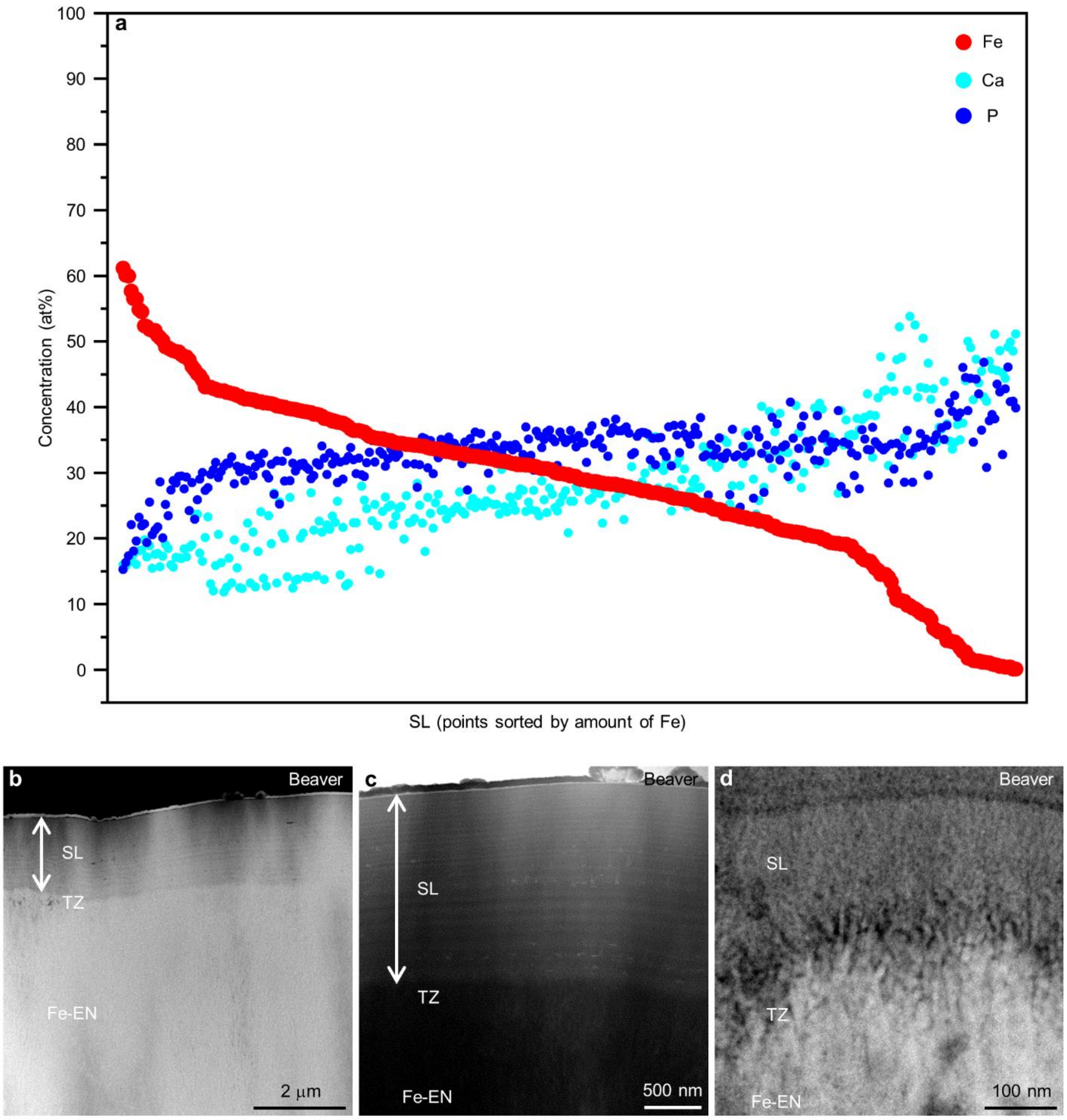


**Figure S10.** Composition of the SL. (a) Fe, Ca and P concentration measurements obtained from EDX spectra measured from 338 positions within the SL. Evaluated and quantified points from the SL of all investigated species are combined and sorted by Fe concentration. Anti-correlation behavior between Fe concentration compared to Ca and P concentrations. (b-d) HAADF-STEM

(b and d) and BF-STEM (c) images of the SL covering the beaver incisor, showing thinner lines oriented parallel to the surface. EDX measurements reveal only minor compositional variations.

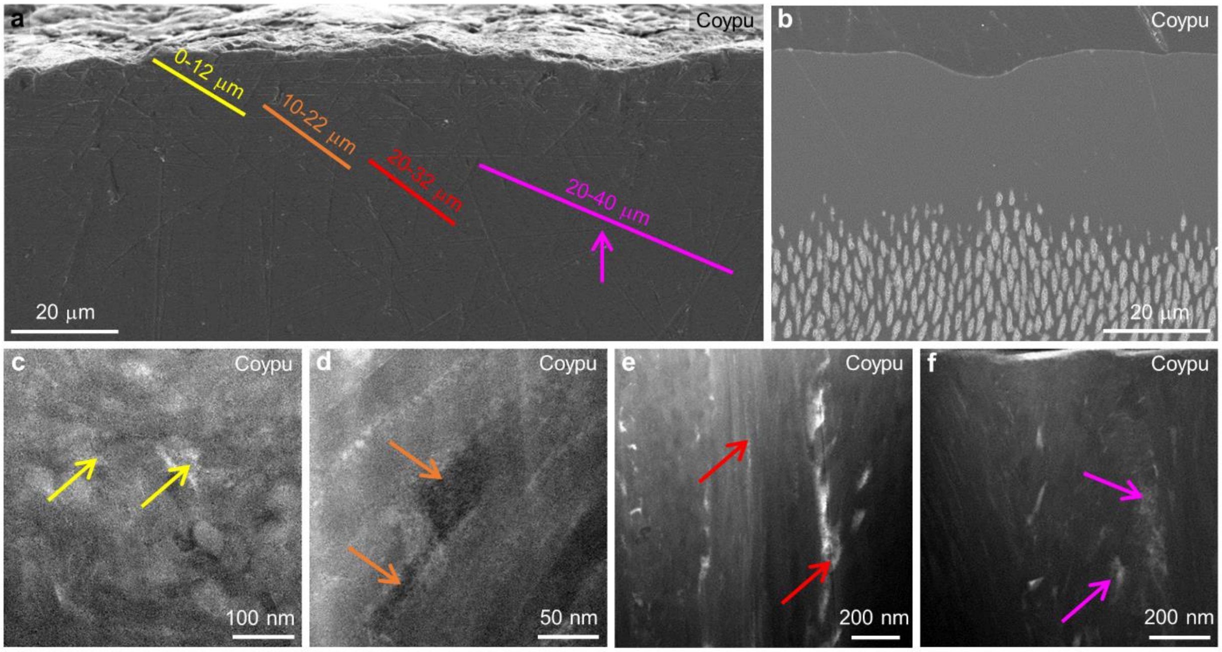


**Figure S11.** TEM investigations of Fe-EN at different distances from the surface. (a) Side view of polished incisor with marked positions of FIB samples prepared in plan-view orientation. The specimens were prepared at different distances from the surface in Fe-EN of coypu incisor. (b) SEM image of etch-polished side view of coypu incisor. (c-f) BF-STEM images taken from the specimens prepared from the positions marked in (a). Arrows mark the positions of pockets filled with ferrihydrite-like material in Fe-EN. The color of the arrows corresponds to the color of the marked positions in (a).

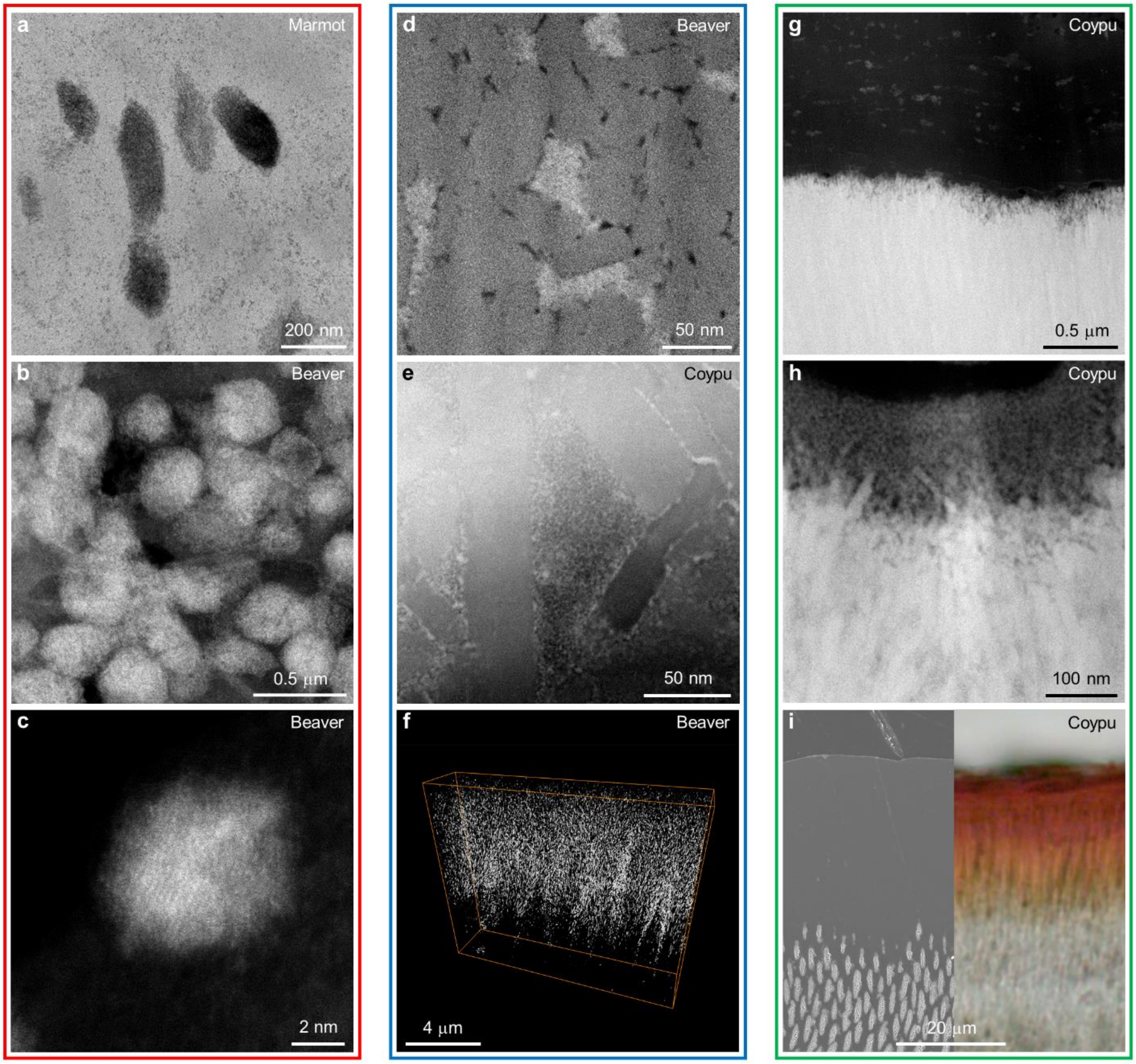


**Figure S12.** Evolution of rodent incisors from late pigmentation stage ameloblasts to Fe-EN and SL. (a-c) (red) Pigmentation stage ameloblasts filled with ferritin nanoparticles. TEM (a) and HAADF-STEM (b) images of ameloblasts filled with ferritin. (c) Atomically resolved HAADF-STEM image of single ferritin nanoparticle. (d-f) (blue) Fe-EN composed of elongated HA crystals and pockets filled with ferrihydrite-like material. HAADF-STEM (d) and BF-STEM (e) images of top-view prepared samples of Fe-EN. (f) 3D reconstruction showing the spatial distribution of filled pockets in Fe-EN. g-i (green) SL, TZ, and Fe-EN. (g) HAADF-STEM image of the SL

attached on the Fe-EN from the non-erupted part of the incisor. (h) Enlarged view of the interface between the SL and Fe-EN showing smaller crystals extending from the rough surface of the enamel covered with SL material. (i) SEM image of the etched side-view of the incisor showing the acid-resistant Fe-EN and the transition to IR-EN (left). Optical micrograph of the same area (right) reveals a special effect, where the color is transmitted from the surface only through the thickness of the Fe-EN.

**Supporting Table**

**Table S1.** Thickness of total EN, and ratios of R-EN vs. total EN and Fe-EN vs. R-EN. Thickness values of total EN, Fe-EN and R-EN were measured from SEM images shown in Figure S6 for all species examined. The ratios appear to be species specific. Although the absolute values for upper and lower incisors are different (coypu and squirrel), the ratios remain the same.

| | Total EN (μm) | R-EN : Total EN | Fe-EN : R-EN |
|---|---|---|---|
| Beaver | 223 | ~ 1:2 | ~ 1:11 |
| Coypu upper | 370 | ~ 1:6 | ~ 1:2 |
| Coypu lower | 323 | ~ 1:6 | ~ 1:2 |
| Marmot | 222 | ~ 1:2 | ~ 1:9 |
| Squirrel upper | 61 | ~ 1:2 | ~ 1:1.5 |
| Squirrel lower | 54 | ~ 1:2 | ~ 1:1.5 |
| Rat | 158 | ~ 1:5 | ~ 1:2 |
| Mouse | 74 | ~ 1:4 | ~ 1:2 |
| Vole | 76 | ~ 1:2 | ~ 1:3 |

## Supporting Movies

**Movie S1:** Fe-EN and 3D spatial arrangement of ferrihydrite-like pockets. The 3D reconstruction of filled ferrihydrite-like pockets within Fe-EN in incisors of beaver at low magnification.

**Movie S2:** Fe-EN and 3D spatial arrangement of ferrihydrite-like pockets. The 3D reconstruction of filled ferrihydrite-like pockets within Fe-EN in incisors of beaver at medium magnification.

**Movie S3:** Fe-EN and 3D spatial arrangement of ferrihydrite-like pockets. The 3D reconstruction of filled ferrihydrite-like pockets within Fe-EN in incisors of beaver at high magnification.
